# Identification of Tumor Antigens and Design of mRNA Vaccine for Colorectal Cancer Based on the Immune Subtype

**DOI:** 10.3389/fcell.2021.783527

**Published:** 2022-01-20

**Authors:** Cong Liu, Dimitri Papukashvili, Yu Dong, Xingyun Wang, Xing Hu, Nuo Yang, Jie Cai, Fengfei Xie, Nino Rcheulishvili, Peng George Wang

**Affiliations:** ^1^ School of Medicine, Southern University of Science and Technology, Shenzhen, China; ^2^ Department of Biomedical Engineering, Southern University of Science and Technology, Shenzhen, China

**Keywords:** mRNA vaccine, colorectal cancer, tumor immune microenvironment, tumor antigens, immune subtype, immune landscape

## Abstract

mRNA vaccines have become a promising alternative to conventional cancer immunotherapy approaches. However, its application on colorectal cancer (CRC) remains poorly understood. We herein identified potential antigens for designing an effective mRNA vaccine, further to build an immune landscape for the accurate selection of patients for mRNA vaccine therapy. Raw transcriptome data from The Cancer Genome Atlas (TCGA) and Gene Expression Omnibus (GEO) databases were retrieved. Consensus clustering algorithm was applied to divide the CRC samples into four immune subtypes. Immunogenomics analysis was further integrated to characterize the immune microenvironment of each immune subtype. Six tumor antigens were found to be associated with poor prognosis and infiltration of antigen-presenting cells (APCs) in CRC patients. Furthermore, each of the immune subtypes showed differential cellular and molecular features. The IS2 and IS4 exhibited significantly improved survival and higher immune cell infiltration compared with IS1 and IS3. Immune checkpoint molecules and human leukocyte antigen also showed significant differential expression in four immune subtypes. Moreover, we performed graph structure learning-based dimensionality reduction to visualize the immune landscape of CRC. Our results revealed a complex immune landscape that may provide directions for mRNA vaccine treatment of CRC and define appropriate vaccination patients.

## Introduction

Colorectal cancer (CRC) is the third most frequently diagnosed tumor, with the second highest mortality rate worldwide (Sung et al., 2021). Currently, effective treatments for CRC mainly include surgical resection, chemotherapy, and radiotherapy (van der Stok et al., 2017). However, morbidity and mortality remain high as approximately 80% of CRC patients show recurrence during the first 3 years (Augestad et al., 2017). Thus, new therapeutic strategies are needed to enhance the survival rate of patients with CRC.

To date, cancer immunotherapies, such as immune checkpoint blockade (ICB) and chimeric antigen receptor T (CAR-T) cell therapy have gained tremendous success (Hargadon et al., 2018; Pantin and Battiwalla, 2020). Messenger RNA (mRNA) vaccine has recently represented a promising alternative to conventional immunotherapy for anti-cancer treatments. *In vitro* transcribed (IVT) mRNA-based gene therapy was initially considered to be an unsuitable approach due to its high immunogenicity and instability. In recent years, along with the development of mRNA synthesis, chemical modification of mRNA and advancing the technology of delivery systems have greatly improved mRNA stability and translation efficacy. IVT mRNA can be designed to express proteins transiently by ensuring that its structure resembles the natural mRNA (Sahin et al., 2014; Kowalzik et al., 2021). Moreover, immunogenicity has gradually become controllable that is of great application prospect in tumor immunotherapy and other biological treatments (Kallen et al., 2013). Compared with the currently available drugs, the mRNA-based approach has a number of advantages: 1) mRNA is produced and purified *in vitro*, without any need of the complex process of protein drug and viral vector preparation; 2) IVT mRNA production process is highly versatile. The production of different target proteins is feasible, hence, time-saving for drug development and efficiency improvement; 3) mRNA only needs to enter the cytoplasm to be translated into protein without entering the nucleus, so no gene insertion and integration take place that improves the safety of the drug (De Keersmaecker et al., 2020); 4) The half-life can be changed by modulating the sequence and the delivery vector (Pardi et al., 2018). mRNA encoding tumor antigens transported via lipid nanoparticle enters into the antigen presenting cells (APCs) and is expressed into the targeted antigen. The expressed tumor antigens can be presented on the surface of APCs by major histocompatibility complexes (MHC) to evoke an anti-tumor response (Miao et al., 2021). Clinical trials have demonstrated that although the protein expression of mRNA is transient, it is effective for tumor immunotherapy applications. In the clinical trials, mRNA vaccines have been applied to treat solid tumors, including non-small cell lung cancers, melanoma, prostate cancer, and glioblastoma (Sebastian et al., 2014; Kubler et al., 2015; Papachristofilou et al., 2019; Batich et al., 2020). mRNA cancer vaccines are combined with ICB to further improve antitumor efficacy (Miao et al., 2021). A great deal of clinical trials has been started and consistently verified the feasibility and suitability of mRNA vaccines for cancer treatment.

Due to the high heterogeneity of CRC at the genetic and molecular level, which affects the efficacy of immunotherapy, there is no mRNA vaccine developed against CRC to date. The effectiveness of mRNA vaccines critically depends on making the antigen(s) of interest available to professional APCs.

The present study aims to predict the potential tumor antigen of CRC for designing an mRNA vaccine. Furthermore, the anti-CRC mRNA cancer vaccine is designed and the immune subtype for identifying suitable CRC patients for vaccination is also explored. Each of the four immune subtypes was correlated with different molecular, cellular features, and clinical outcomes. Our results might provide the theoretical basis of developing an mRNA cancer vaccine and facilitate the selection of optimal CRC patients for vaccination.

## Materials and Methods

### Patients and Datasets

The RNA sequencing (RNA-seq) gene expression data of 612 CRC samples and their clinicopathologic information were downloaded from TCGA database (https://gdc-portal.nci.nih.gov/) as our discovery cohort. RNA-seq data of 598 CRC samples (GSE39582) and the clinical information were downloaded from the Gene Expression Omnibus (GEO) database (https://www.ncbi.nlm.nih.gov/geo/). An independent cohort GSE39582 was used for further validation. Building on TCGA datasets, gene mutation information was obtained via secondary databases like cBioportal (http://www.cbioportal.org/). To discover unbiased immune subtypes, we identified 1,989 IRGs in five different categories including single-cell RNA-seq data, the gene of co-stimulatory and co-inhibitory molecules, the gene of cytokine and cytokine receptors, genes involved in antigen processing and presentation, and other immune-related genes (Chen et al., 2019; Li et al., 2019).

### cBioPortal Database Analysis

The cBioPortal (http://www.cbioportal.org/) was used to analyze and visualize cancer genomics data sets including copy number variation and mutation (Cerami et al., 2012). The genetic alterations of potential tumor antigens against tumors were obtained from cBioPortal based on 612 CRC samples in TCGA.

### GEPIA Database Analysis

The online database Gene Expression Profiling Interactive Analysis (GEPIA2) (http://gepia2.cancer-pku.cn/) which is based on samples from the TCGA and the GTEx (Genotype-Tissue Expression) databases was used for gene expression analysis to further clarify the relationship between tumor antigen gene expression and CRC prognosis (Tang et al., 2017). ANOVA was used for differential expression analysis to compare the tumor to paired normal samples. The chromosomal distribution of over- or under-expressed genes were plotted in differential genes with |Log2FC| > 1 and q-value < 0.01. Kaplan–Meier estimates of OS and RFS were performed to evaluate the prognostic value of identified tumor antigens.

### TIMER Database Analysis

To assess the correlation between the infiltration level of APCs and the expression of the identified tumor antigens TIMER database was used. (https://cistrome.shinyapps.io/timer/). TIMER includes 10,897 samples across 32 cancer types from TCGA to estimate the abundance of immune infiltrates. Prognostic gene expression data was used to calculate the abundance of six immune cell types (B cells, CD4+T cells, CD8+T cells, neutrophils, macrophages, and dendritic cells) in CRC patients using the TIMER (Li et al., 2017).

### Discovery and Validation of the Immune Subtypes

Based on the expression of 1,989 IRGs, the 201 IRGs impacting prognosis of CRC patients by univariate Cox regression analysis was performed in subsequent analysis. Consensus clustering analysis was used to identify the four immune subtypes by using the R package “Consensus Cluster Plus” with parameters defined as a maximum of nine clusters. To assess the robustness of the identified immune subtypes, we further conducted the same algorithm in the validation cohort (GEO). In-group-proportion statistic was calculated to validate the similarity of immune subtypes across the discovery and validation cohorts.

### Assessing the Clinicopathological, Cellular and Molecular Characteristics Associated With the Immune Subtypes

The correlation between immune subtypes and clinicopathological characteristics was evaluated by the Chi-square test. The prognostic value of the immune subtypes with OS as the endpoint was analyzed by the log-rank test and multivariable Cox proportional hazards regression model. The ANOVA algorithm was performed to assess the correlation between immune subtypes and different immune-related molecular and cellular characteristics. Finally, we employed the ssGSEA algorithm via R packages “GSVA” to comprehensively calculate the enrichment score of CRC samples on each immune-related term.

### Defining the Immune Landscape

Considering the dynamic nature of the immune system, the dimensionality reduction analysis using the graph learning-based method was performed to reveal the intrinsic structure and visualize the distribution of individual patients (Trapnell et al., 2014). The discriminative dimensionality reduction with trees (DDRTree) was performed for dimension reduction (Qiu et al., 2017). Finally, the immune landscape was visualized with the function plot cell trajectory (Monocle) with the color corresponding to the immune subtype identified above (Qi et al., 2017; Wang and Mao, 2019).

### Statistical Analysis

All statistical analyses and data visualization were performed in R version 4.0.5, and GraphPad Prism 8.0. *p* < 0.05 was considered statistically significant.

## Results

### Identification of Potential Tumor Antigens in CRC

**FIGURE 1:**
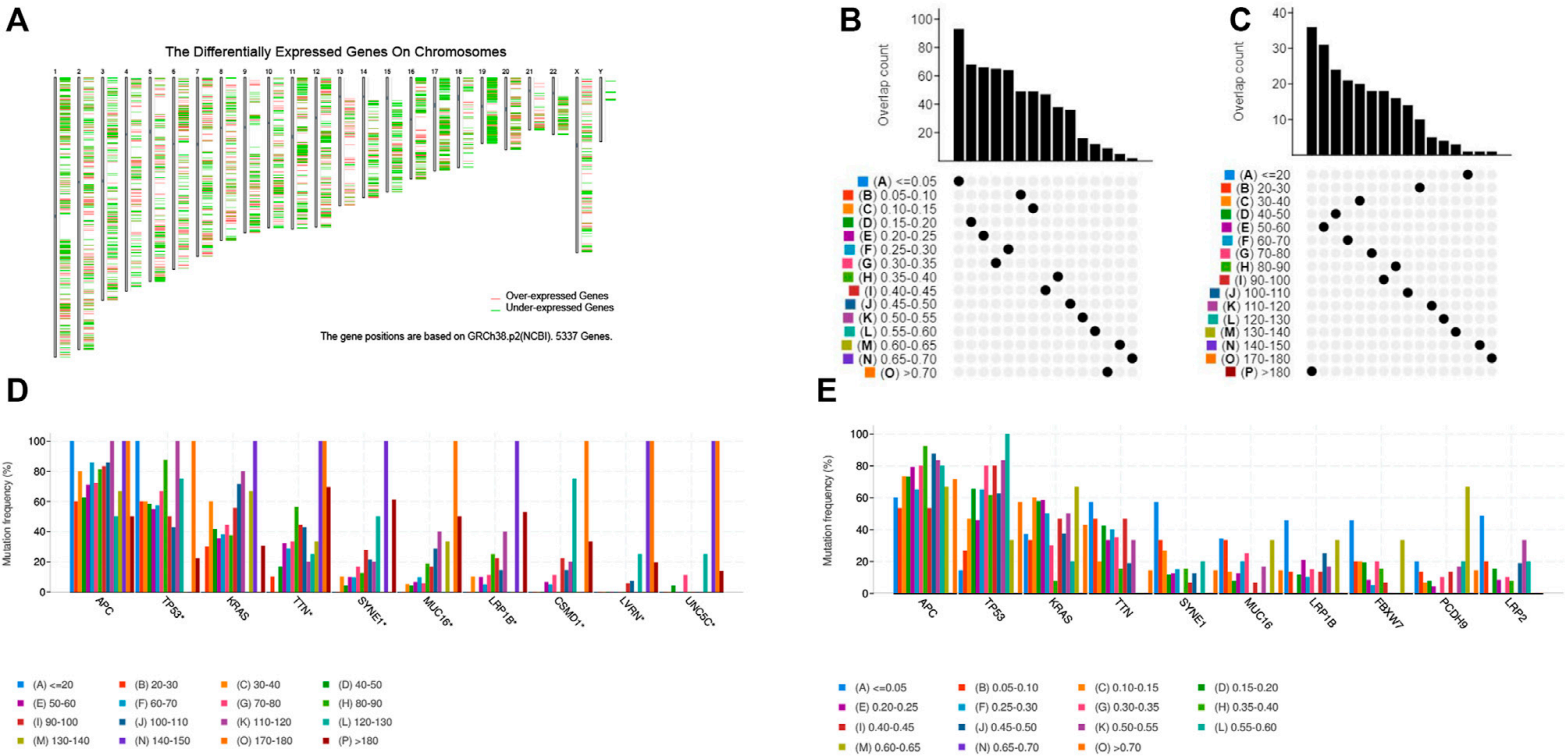
Identification of potential tumor-associated antigens of CRC. **(A)** Chromosomal distribution of up-regulated and down-regulated genes in CRC. **(B)** Overlapping samples in altered genome fraction group. **(C)** Overlapping samples in mutation count group. **(D)** Highest frequency genes in mutation count group. **(E)** Highest frequency genes in altered genome fraction group.

To identify potential tumor antigens of CRC, 2658 over-expressed genes that could potentially encode tumor-associated antigens (TAA) were detected by screening for aberrantly expressed genes (Figure 1A). In addition, 14,751 mutated genes encoding TAA were filtered by analyzing fraction of genome altered and tumor mutational count in CRC patients (Figures 1B,C). Mutational analysis showed that the adenomatous polyposis coli (APC), tumor suppressor P53 (TP53), KRAS proto-oncogene GTPase (KARS), titin (TTN), spectrin repeat containing nuclear envelope protein 1 (SYNE1), mucin 16 (MUC16), and low-density lipoprotein receptor-related protein 1B (LRP1B) were the most frequently mutated genes in the fraction genome alteration and tumor mutational count group (Figures 1D,E). Collectively, we identified 2052 over-expressed and mutated genes.

### Correlation of Tumor Antigens with CRC Prognosis and APCs for mRNA Vaccine Design

**FIGURE 2:**
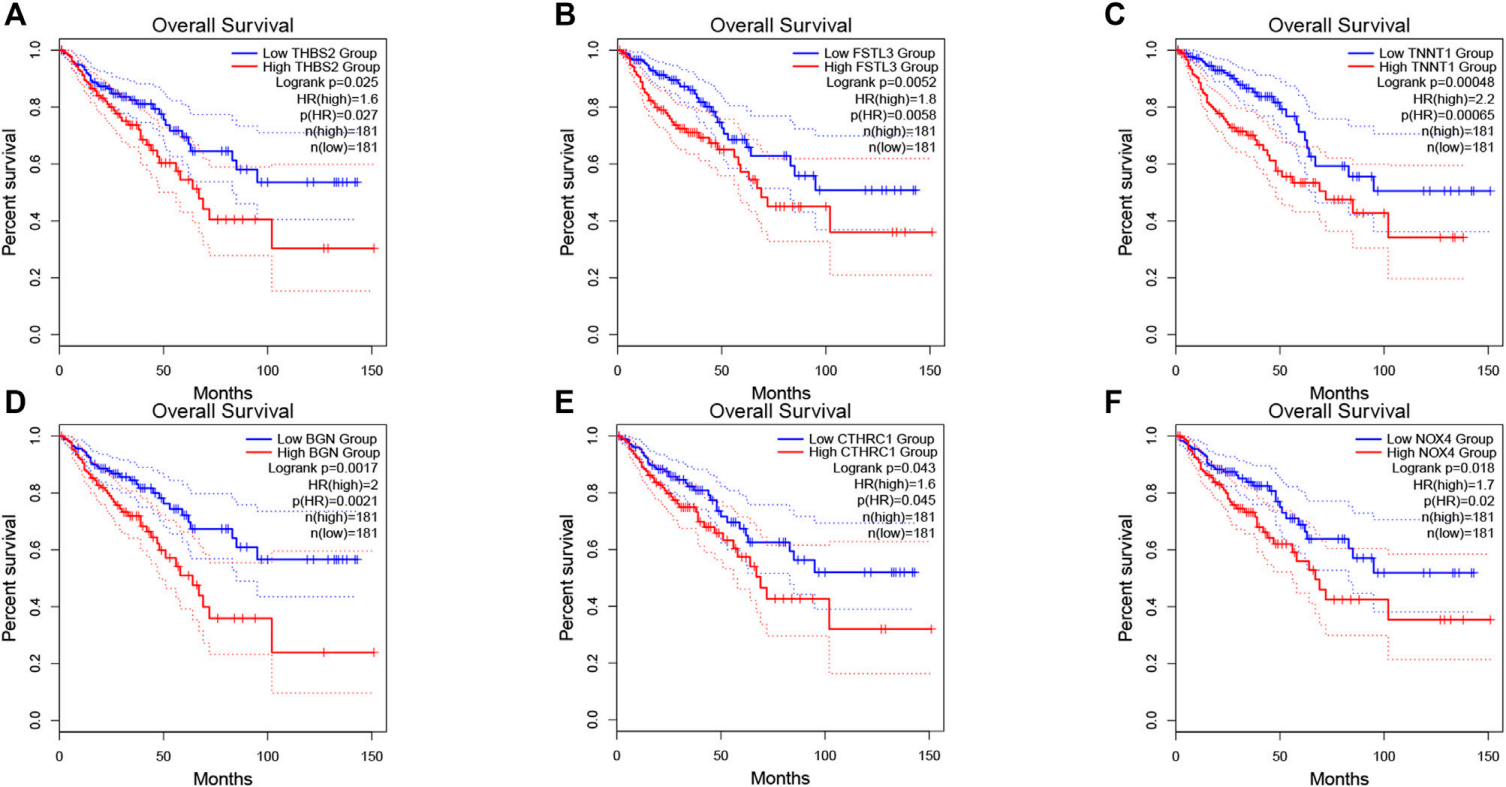
Correlation of tumor antigens with CRC prognosis. **(A–F)** The association of THBS2 **(A)**, FSTL3 **(B)**, TNNT1 **(C)**, BGN **(D)**, CTHRC1 **(E)**, and NOX4 **(F)** expression levels with the OS of CRC patients. The red line represents the samples with highly expressed genes and the blue line indicates the samples with lowly expressed genes.

**FIGURE 3:**
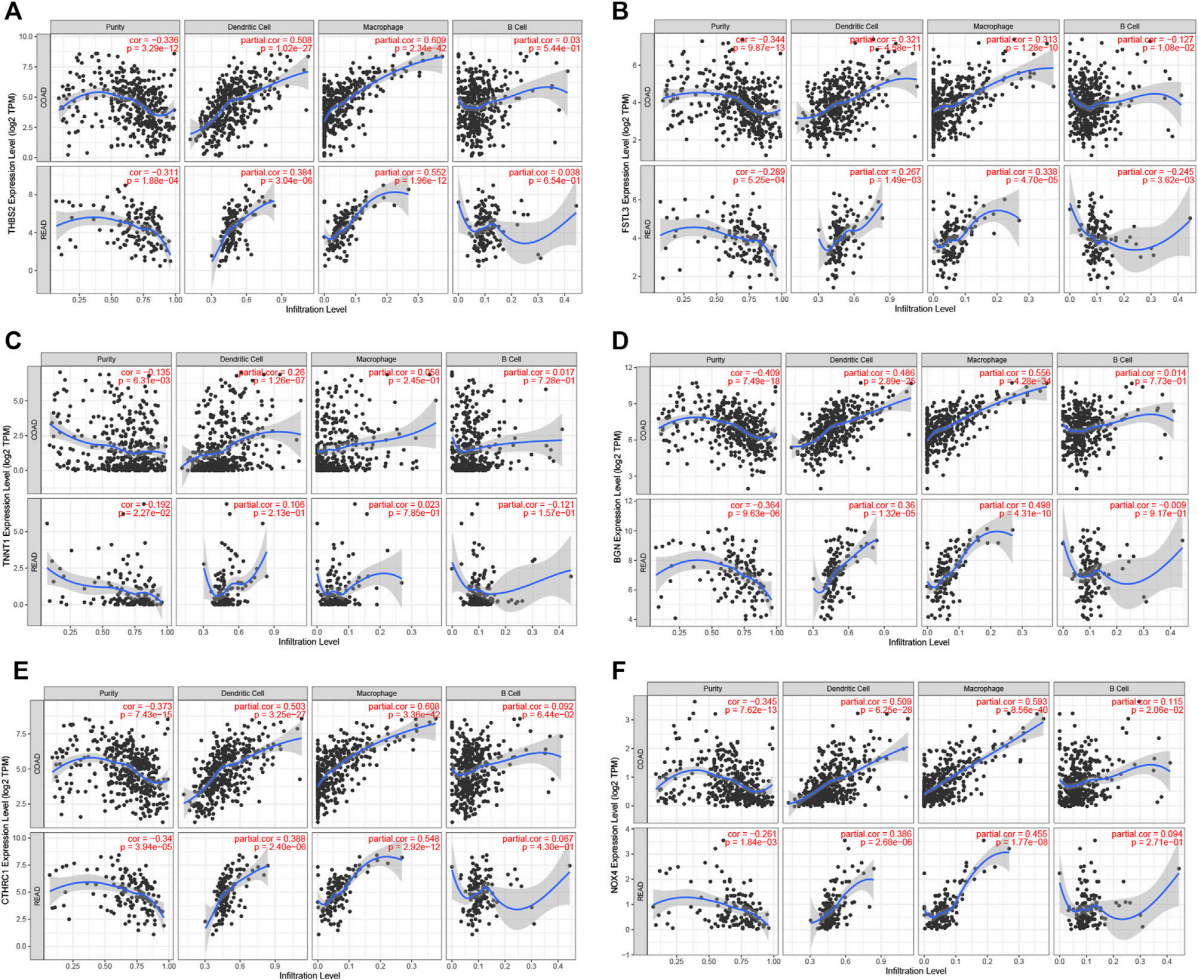
Correlation of tumor antigens expression with antigen-presenting cells level in CRC. **(A–F)** Correlation between THBS2 **(A)**, FSTL3 **(B)**, TNNT1 **(C)**, BGN **(D)**, CTHRC1 **(E)**, and NOX4 **(F)** expression and the level of infiltrating immune cells dendritic cells, macrophages, and B cells in CRC.

**FIGURE 4:**
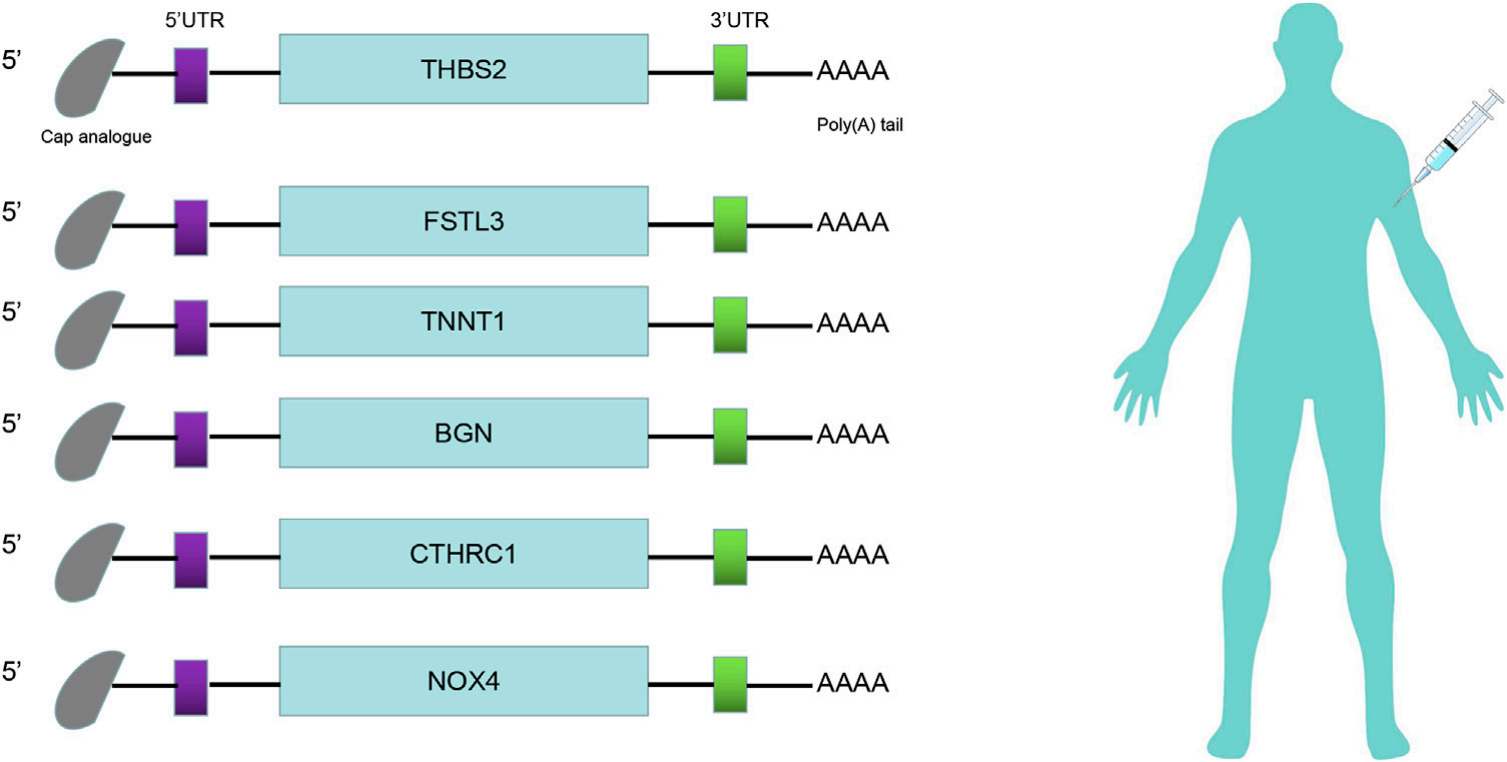
mRNA-vaccine design. Structure of the anti-CRC mRNA vaccine. The 5′-cap analog, 5′- and 3′-untranslated regions (UTRs), and poly(A) tail were optimized for stability and translational efficiency.

The prognosis-associated tumor antigens were selected from the above-mentioned genes as promising candidates for designing the mRNA vaccine. Fifty-one genes were strongly correlated with the overall survival (OS) of CRC. Only six genes were closely related to the disease-free survival (RFS) (Supplementary Figure S1). The survival analysis revealed that elevated expression of thrombospondin 2 (THBS2), follistatin like 3 (FSTL3), troponin T1 (TNNT1), biglycan (BGN), collagen triple helix repeat containing 1 (CTHRC1), and NADPH oxidase 4 (NOX4) were associated with poor OS in CRC (Figures 2A–F). In addition, we found a shorter RFS time in patients with higher expression levels of THBS2, FSTL3, TNNT1, BGN, CTHRC1, and NOX4 (Supplementary Figure S2). The effectiveness of mRNA vaccines strongly relies on the availability of the target antigen(s) to professional APCs, especially dendritic cells (DCs), macrophages, and B cells. Therefore, we investigated whether the expression of THBS2, FSTL3, TNNT1, BGN, CTHRC1, and NOX4 was correlated with immune cell infiltration levels in CRC via the Tumor Immune Estimation Resource (TIMER) database. The results demonstrated that elevated expression of THBS2, FSTL3, BGN, CTHRC1, and NOX4 was exhibited in increased infiltration of macrophages, DCs and B cells in CRC, although it was insignificant in B cells. Additionally, TNNT1 expression has the same tendency of increased infiltration of macrophages, DCs, and B cells (Figures 3A–F). Taken together, we designed mRNA encoding the antigen of interest flanked by 5′ and 3′ untranslated regions (UTRs). As shown in Figure 4, six tumor antigens (THBS2, FSTL3, TNNT1, BGN, CTHRC1, and NOX4) were identified as potential therapeutic candidates for the anti-CRC mRNA vaccine that can be processed and presented by APCs to activate a robust immune response.

### Immune Subtypes of CRC

**FIGURE 5:**
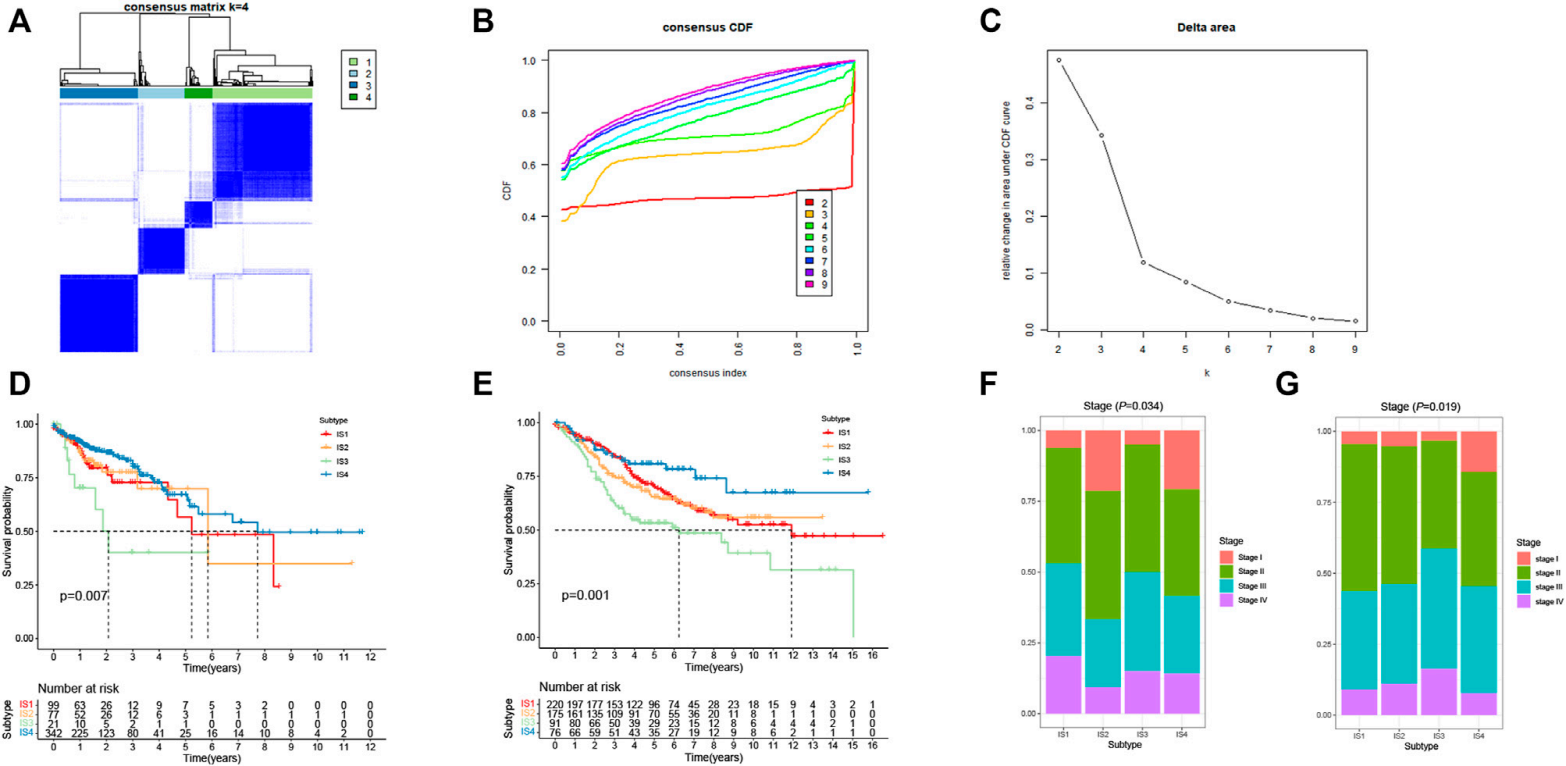
Identification of immune subtypes of CRC. **(A)** Consensus clustering matrix of CRC samples for *k* = 4. **(B)** Cumulative distribution function (CDF) curve of the consistency score for different immune subtype numbers (*k* = 2–9). **(C)** Delta area plot showing the relative change in area under the CDF curve from *k* = 2 to *k* = 9, which assisted in determining the optimal immune subtype numbers. **(D)** Kaplan-Meier survival curves of CRC immune subtypes in TCGA cohort. **(E)** Kaplan-Meier survival curves of CRC immune subtypes in GEO cohort. **(F)** Stages of CRC patients among the IS1-IS4 immune subtypes in TCGA cohort. **(G)** Stages of CRC patients among the IS1-IS4 immune subtypes in GEO cohort.

Immunophenotyping can be used to reflect the immune status of the tumor microenvironment, thus, allow the selection of patients for mRNA vaccine treatment. Therefore, consensus clustering was performed in 568 CRC samples based on the immune-related genes (IRGs). The results were visualized using a cumulative distribution function (CDF) plot and a delta area plot, in which k represents the number of immune subtypes. Four robust immune subtypes (IS1-IS4) were identified and the IRGs appeared to be stably clustered when *K* = 4 (Figures 5A–C). Significantly prognostic impact of the immune subtypes in TCGA cohort was observed. Immune subtypes IS2 and IS4 were associated with a better prognosis for OS. Conversely, patients in IS1 and IS3 displayed the poorer survival probability in the TCGA cohort (Figure 5D). Consistent with our results obtained from the TCGA cohort, each of the immune subtypes was related to prognosis in the GEO cohort as well (Figure 5E). Among all immune subtypes, IS2 and IS4 were associated with the lower stage (Figures 5F,G) compared with IS1 and IS3. Taken together, immunophenotyping could be a robust prognostic indicator and its accuracy is superior to conventional stage of the tumor in CRC.

### Correlation of Immune Subtypes with Tumor Mutational Burden

**FIGURE 6:**
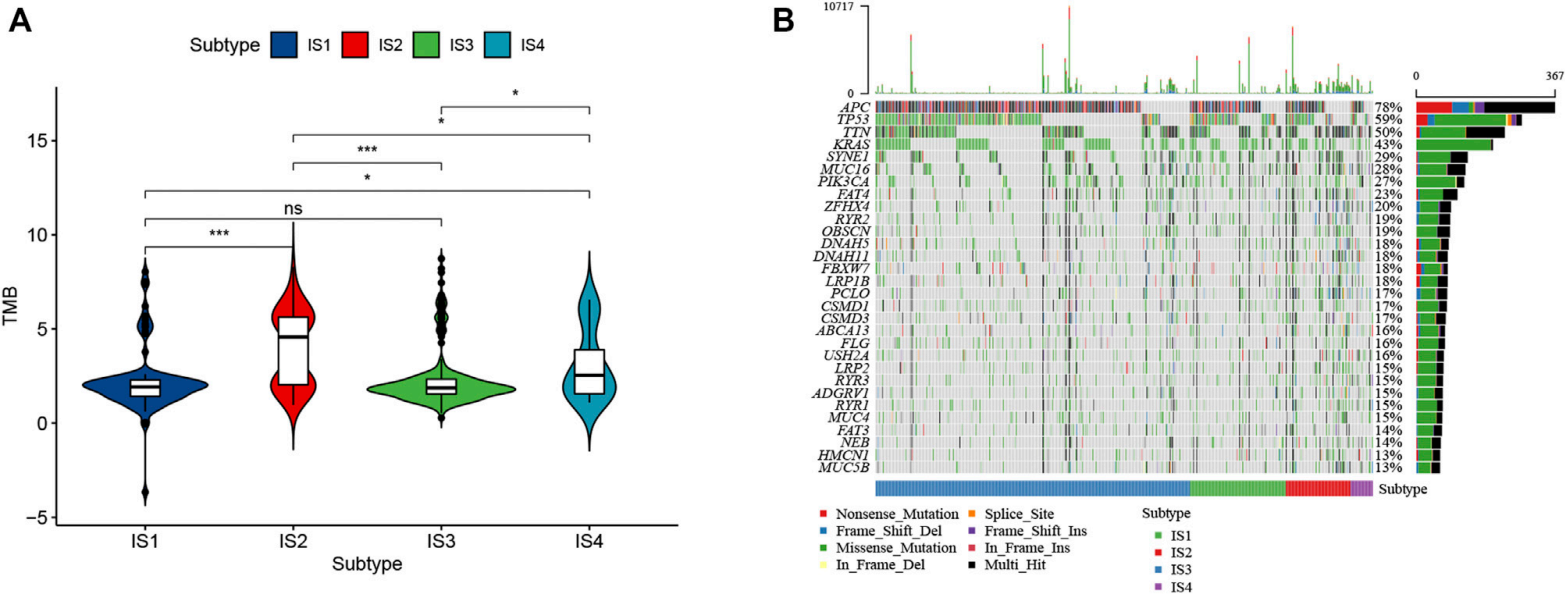
Correlation of immune subtypes with TMB in CRC. **(A)** The TMB of CRC samples from four immune subtypes. **(B)** The alteration landscape of CRC across four immune subtypes.

High tumor mutational burden (TMB) can increase the tumor immunogenicity, allow the immune system to recognize and attack the cancer cells by T cell-mediated antitumor immunity. Therefore, TMB for each patient can be detected and calculated using the VarScan method in the TCGA cohort. Subtypes IS2 and IS4 had significantly higher TMB than subtypes IS1 and IS3 (Figure 6A). Furthermore, somatic mutation profiling revealed a high frequency of mutations in APC (78%), TP53 (59%), TTN (50%), KRAS (43%), SYNE1 (29%), and MUC16 (28%) across immune subtype (Figure 6B). These findings suggest that the immune subtype is a promising tool to predict TMB in CRC patients and clinical response to mRNA vaccine. Therefore, patients belong to IS2 and IS4 may respond positively to the mRNA vaccine.

### Correlation of Immune Subtypes with Immune Checkpoint Molecules and HLA

**FIGURE 7:**
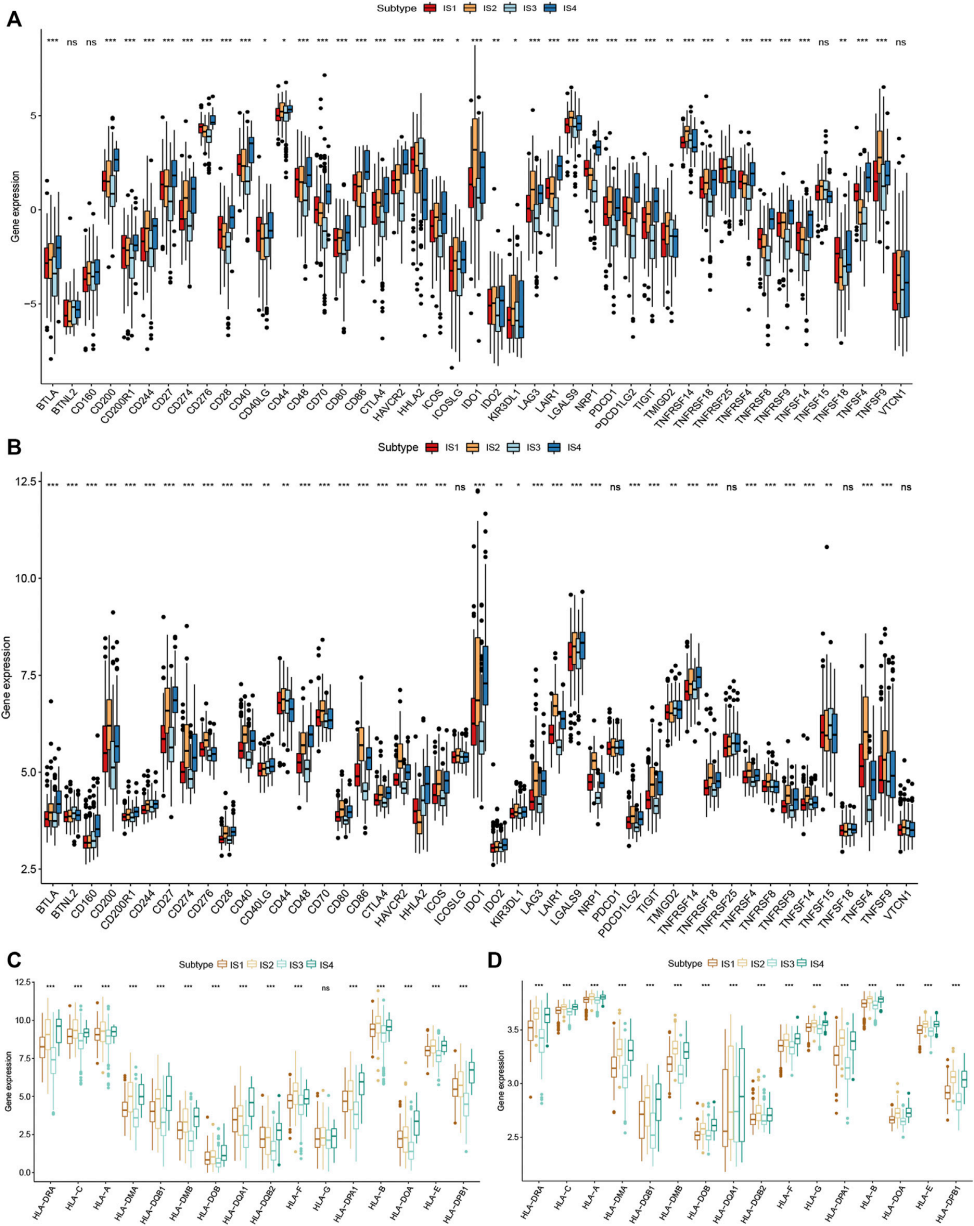
Correlation of immune subtypes with immune checkpoint molecules and HLA genes expression in CRC. **(A)** The expression of immune checkpoint molecules among CRC immune subtypes in TCGA cohort. **(B)** The expression of immune checkpoint molecules among CRC immune subtypes in GEO cohort. **(C)** The expression of HLA genes among the CRC immune subtypes in TCGA cohort. **(D)** The expression of HLA genes among the CRC immune subtypes in GEO cohort. *p < 0.05, **p < 0.01, and ***p < 0.001.

Immune checkpoint molecules and human leukocyte antigen (HLA) are essential for immune function and have diverse clinical implications in cancer immunotherapy. Therefore, we next analyzed the relationship between the CRC immune subtypes and the expression level of immune checkpoint molecule genes and HLA. We found that 30 immune checkpoint-related genes were significantly differentially expressed among the immune subtypes. For example, TNFSF4, TNFRSF9, TNFRSF4, TNFRSF18, LAG3, JAK2, PTPRC, IL12B, IFNG, ICOS, HAVCR2, CTLA4, CD8A, CD86, CD80, CD40, CD28, and CD274 were significantly downregulated in IS3 tumors, while TNFSF9, TNFSF4 TNFRSF9, TNFRSF4, TNFRSF18, PTPRC, CTLA4, CD8A, CD86, CD80, CD40, CD28, and CD274 were strongly elevated in IS2 tumors (Figures 7A,B). Next, we assessed the expression level of HLA genes in the four immune subtypes and determined that the expression levels were significantly elevated in IS2 and IS4 than in IS1 and IS3 (Figures 7C,D). HLA plays important role in the activation of anti-tumor immunity through interactions with T cell receptors. Thus, the response of patients with IS2 and IS4 on mRNA vaccine treatment might be more promising.

### Cellular and Molecular Features of CRC Immune Subtypes

**FIGURE 8:**
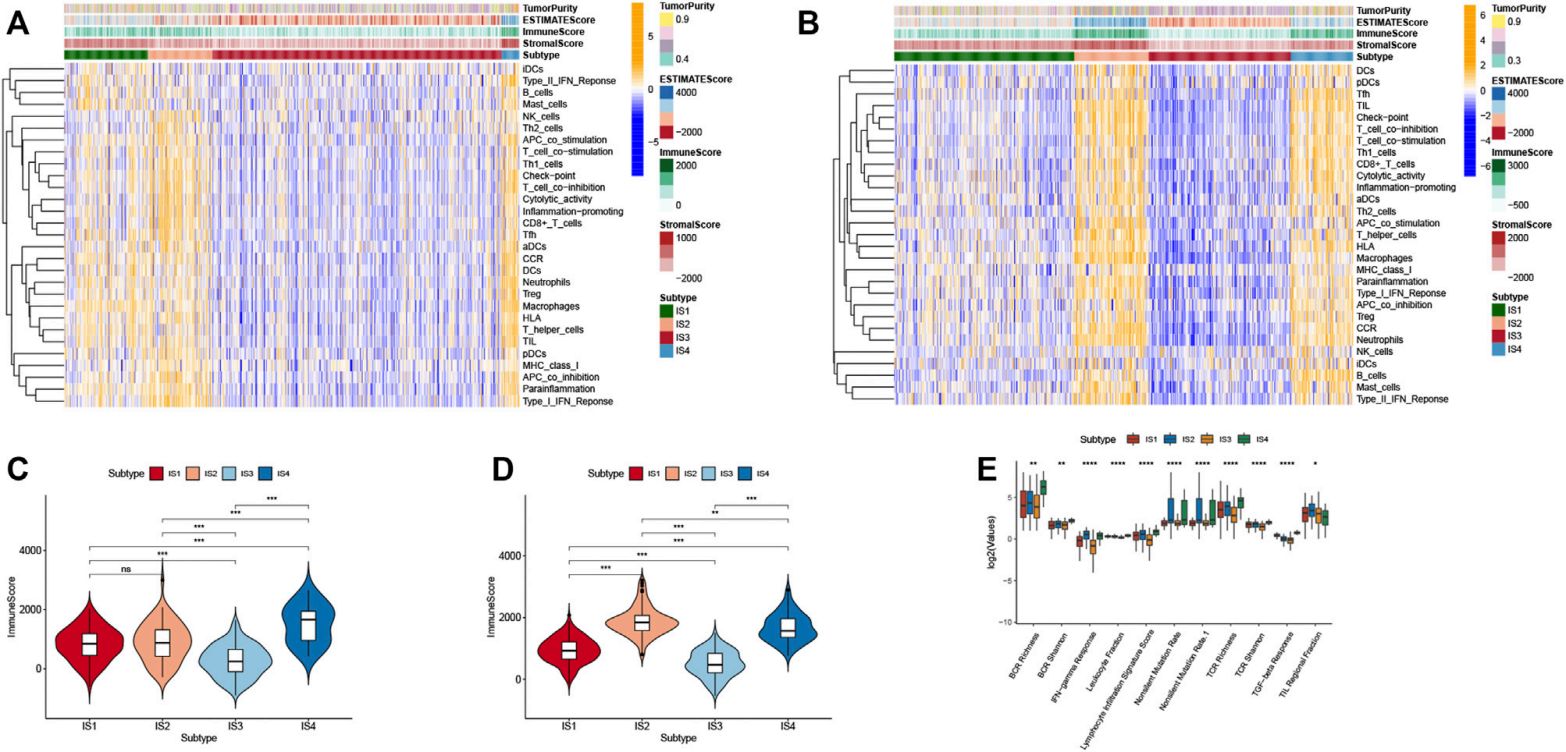
Cellular and molecular features of immune subtypes. **(A)** The enrichment levels of 29 immune-related gene sets among CRC immune subtypes in TCGA cohort. **(B)** The difference in Immune Scores between four immune subtypes in TCGA cohort. **(C)** The enrichment levels of 29 immune-related gene sets among CRC immune subtypes in GEO cohort. **(D)** The difference in Immune Scores between four immune subtypes in GEO cohort. **(E)** The enrichment levels of 11 immune-related gene sets among CRC immune subtypes.

The tumor immune microenvironment (TIME) plays a crucial role in CRC progression, thus influences the effectiveness of the mRNA vaccine. We assessed the relationship between the immune subtypes and 29 immune-related molecular features in the TCGA and GEO cohorts by the single-sample gene set enrichment analysis (ssGSEA) algorithm (Figures 8A,B). The results showed that IS2 and IS4 have significantly higher levels of B cells, CD+8 T cells, Th1 cells, and macrophages compared with IS1 and IS3. Moreover, we calculated stromal and immune scores to quantify the abundances of infiltrating stromal and immune cells for each sample in TCGA and GEO. These results also demonstrated that IS2 and IS4 have higher Immune Score than IS1 and IS3 (Figure 8C). Thus, IS2 and IS4 are immune-hot phenotypes, while IS1 and IS3 are immune-cold phenotypes. Similar results were also validated in patients in the GEO cohort (Figure 8D).

To better understand the association of CRC immune subtypes with six pan-cancer immunotypes we extracted 11 immune-related molecular features from a previous study and demonstrated that the expression of 11 molecular signatures was significantly different among the four immune subtypes (Figure 8E). Antigen-specific T cell receptor (TCR) and B cell receptor (BCR) repertoires play a significant role in the immune system for the recognition of malignant cells. We observed that IS2 and IS4 had higher scores in TCR richness and BCR richness. Higher TCR richness may enhance anticancer immunity. Therefore, immunotype can be used to reflect CRC immune status and to select suitable patients for mRNA vaccine treatment.

### Immune Landscape of CRC

**FIGURE 9:**
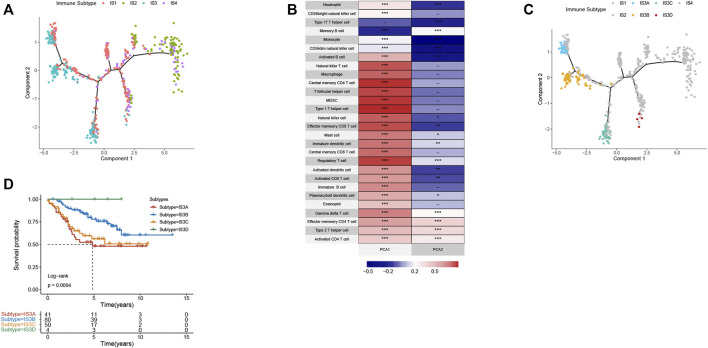
The immune landscape of CRC. **(A)** The immune landscape of CRC. Each point represents a patient with colors corresponding to the immune subtype defined previously. **(B)** Correlation between PCA1/2 and immune cell signatures. **(C)** Immune landscape of the subsets of CRC IS3 based on their location in the immune landscape. **(D)** Different subtypes in IS3 associated with different prognoses.

The immune landscape is increasingly recognized to be important in cancer development, progression, and response to therapy. Subsequently, we sought to visualize the immune landscape by the graph learning-based dimensionality reduction technique. We observed that the immune-hot subtype IS2 and immune-cold subtype IS3 were distributed at the opposite end of the horizontal axis in the immune landscape (Figure 9A). The horizontal axis in the immune landscape may represent the overall immune infiltration. As shown in Figure 9B, the horizontal axis was associated with various immune cells, of which activated CD8+ T cells, activated CD4+ T cells, and type 1 T helper cells showed the positive correlation.

The immune landscape further revealed significant intra-cluster heterogeneity within each subtype. IS3 could be further divided into four subgroups based on the distribution location in the immune landscape (Figure 9C). The further prognostic analysis demonstrated that IS3D has the best prognosis among IS3A, IS3B, and IS3C (Figure 9D). Collectively, the immune landscape based on immune subtypes can precisely recognize immune components of each CRC patient as well as predict their prognoses that are beneficial for selecting personalized therapeutics for mRNA vaccine.

## Discussion

Therapy of CRC has been improved over the years with advanced surgical and chemotherapeutic procedures but challenges in terms of efficiency and adverse effects still need to be accomplished. Especially late-stage CRC patients still have a relatively poor prognosis (Hammond et al., 2016; Russo et al., 2019). mRNA vaccines have become a promising platform for cancer treatment. Nevertheless, tumor heterogeneity is one of the biggest setbacks in implementing mRNA vaccine-based therapy. In this study, the abnormal gene expression patterns and mutational landscape of CRC were constructed and a range of targeted antigens, including THBS2, FSTL3, TNNT1, BGN, CTHRC1, and NOX4 were identified as promising mRNA vaccine candidates. The overexpression of these antigens was associated with poor prognosis and high infiltration of APCs in CRC. Thus, these antigens play important roles in CRC development and progression. According to the abovementioned, the sequence of tumor antigens can be modified and cloned into appropriate plasmids for synthesizing mRNA. IVT mRNA will be encapsulated with lipid nanoparticles. Optimized mRNA encoding CRC antigens will be injected into CRC patients to induce an immune response against tumor cells to eradicate the tumor. mRNA vaccines encoding these tumor antigens (THBS2, FSTL3, TNNT1, BGN, CTHRC1, and NOX4) induce a cell-mediated immune response and humoral immune response that are beneficial for efficient clearance of cancer cells. Although further clinical evaluation of the candidates is required, their potential for anti-CRC mRNA vaccine development is consolidated by previous studies. For instance, THBS2 is a biomarker for clinical stages and a strong prognostic indicator in CRC. Increased THBS2 expression was significantly associated with clinical stages (TNM). Furthermore, THBS2 has been reported to regulate apoptosis, cell proliferation, and adhesion (Wang et al., 2016; Tian et al., 2018). FSTL3 is an oncogene and may be involved in CRC progression via the promotion of bone morphogenetic protein (BMP) signaling pathway (Karagiannis et al., 2013). Similarly, TNNT1 expression was closely correlated with the clinical stage, facilitated the proliferation, migration, and invasion of CRC cells (Chen Y. et al., 2020). BGN has been reported to be implicated in CRC liver metastasis (Ma et al., 2018). BGN activity influences CRC progression via its participation in other pathways (Chen D. et al., 2020). Previous studies have reported that knockout of BGN can inhibit the proliferation and migration of CRC cells (Xing et al., 2015a). In addition, BGN can promote angiogenesis through upregulating vascular endothelial growth factor (VEGF) expression in CRC patients (Xing et al., 2015b; Suhovskih et al., 2015). Moreover, BGN can also serve as a prognostic biomarker in CRC patients (You et al., 2015). CTHRC1 promotes human CRC cell proliferation and invasiveness by activating Wnt/planar cell polarity (PCP) signaling pathway (Yang et al., 2015). Upregulated CTHRC1 predicts poor prognosis and promotes epithelial-mesenchymal transition in CRC (Ni et al., 2018). NOX4 plays an important role in CRC progression and metastasis (Shen et al., 2020). High NOX4 expression was significantly correlated with clinicopathologic classifications and poor survival of CRC patients. NOX4 promoted CRC cells proliferation, migration, and invasion by regulating relevant pathway (Lin et al., 2017). To our knowledge, this is the first study that screened the CRC antigens for the development of mRNA vaccine for cancer.

Given that tumor heterogeneity restricts the widespread application of mRNA vaccine in CRC patients, a comprehensive understanding of the immune landscape of CRC is therefore essential for the design of efficient immunotherapy strategies. Four reproducible immune subtypes of CRC were identified based on IRGs expression profile to provide new insights for designing advanced mRNA vaccine strategies for CRC treatment. We observed that each of the four immune subtypes was correlated with distinct clinical characteristics, and accordingly revealed different patterns in molecular and cellular features. IS2 and IS4 tumors displayed a better prognosis than other subtypes in TCGA and GEO datasets. TMB is a critical intrinsic factor that affects tumor response to immunotherapy. High TMB can be used as an independent biomarker for the selection of patients for mRNA vaccine therapy (Havel et al., 2019). Patients with IS2 and IS4 tumors showed higher TMB, which may have a greater response to mRNA vaccine treatment. Moreover, we found that the IS2 and IS4 tumors had higher expression of HLA and immune checkpoint molecules and showed a more prominent Th1/IFNγ gene signature. HLA expression contributes to the activation of anti-tumor immunity through interactions with T cell receptors. Moreover, the complex immune landscape of CRC revealed substantial heterogeneity between individual patients and the same immune subtype, which provided complementary information for personalized mRNA-based cancer vaccines.

TIME plays a vital role for mRNA vaccine to elicit tumor immune response, it is critical to further understand diverse immune cell components of different immune subtypes. We found that each of the four immune subtypes was related to distinct cellular and molecular features. Among all immune subtypes, IS2 and IS4 have high immune scores compared with IS1 and IS3, which indicated that IS2 and IS4 are immunologically “hot” tumors while IS1 and IS3 are immunologically “cold” tumors. High immune scores subtype (IS2 and IS4) showed better survival outcomes. Previous studies proposed the “hot” tumor displayed higher tumor sensitivity to immunotherapy (Galon et al., 2006; Galon and Bruni, 2019). These findings further showed that patients in the immune-cold subtype have the lowest degree of B cells and CD4+T cells infiltration, while the immune-hot subtype exhibited high levels of infiltrating immune cells. IS2 and IS4 displayed abundant tumor-infiltrating lymphocytes (TILs), cytotoxic T cells, natural killer cells, and macrophages, the high level of TILs recognize ample tumor antigens with their diverse receptors. IS2 and IS4 had higher scores in TCR richness and BCR richness. Higher TCR richness may enhance anticancer immunity (Thorsson et al., 2018). Moreover, IS2 and IS4 exhibited higher inflammation-promoting, IFN-γ response, and TGF-β response gene expression compared with IS1 and IS3. Thus, patients with IS2 and IS4 tumors are more suitable for mRNA vaccine therapy, IS1 and IS3 may respond to mRNA vaccine combined with ICB therapy. However, our study was limited because it was retrospective, and identified mRNA vaccine antigens of the current investigation demand validation by prospective studies. The findings should be interpreted with this limitation in mind. Additionally, we will attempt to validate the accuracy of the identified six antigens in our following work.

In summary, our findings depicted the immune landscape of CRC and eventually identified four immune subtypes. THBS2, FSTL3, TNNT1, BGN, CTHRC1, and NOX4 are the potential targets of the CRC mRNA vaccine. Patients with IS2 and IS4 are suitable candidates for vaccination. In addition, we design an anti-CRC mRNA vaccine. Hence, this study may guide a personalized approach for mRNA vaccine development against CRC.

## Data Availability

Publicly available datasets were analyzed in this study. This data can be found here: TCGA database (https://gdc-portal.nci.nih.gov/), GEO database (https://www.ncbi.nlm.nih.gov/geo/), cBioPortal (http://www.cbioportal.org/), GEPIA2 (http://gepia2.cancer-pku.cn/), and TIMER database https://cistrome.shinyapps.io/timer/.
