# Supplementary figures and images for: Identification of Tumor Antigens and Design of mRNA Vaccine for Colorectal Cancer Based on the Immune Subtype
Source: Front Cell Dev Biol. 2022 Jan 20;9:783527. doi: 10.3389/fcell.2021.783527 (PMC8811447; doi:10.3389/fcell.2021.783527)

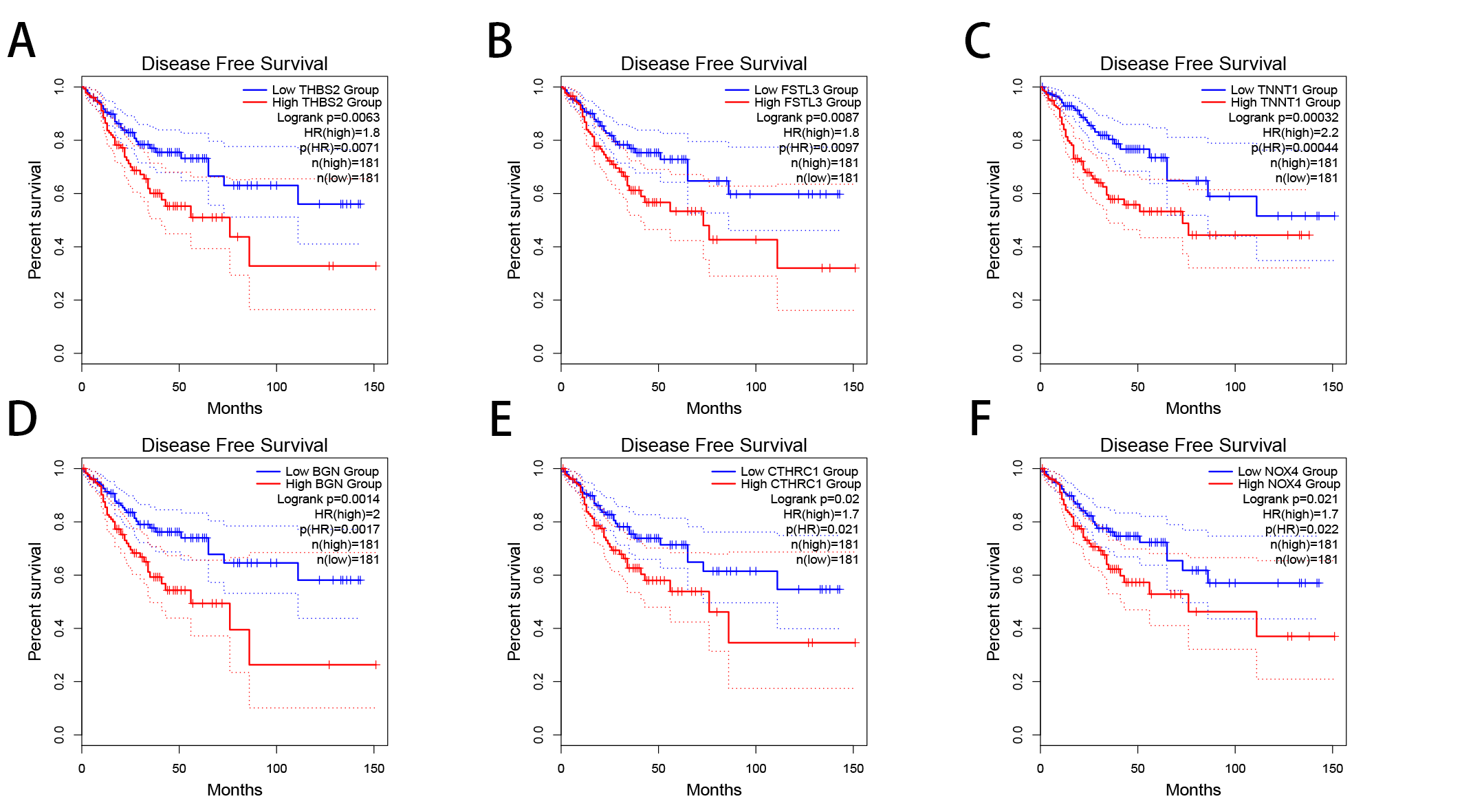

Supplement: Supplementary file 1 [file Image2.TIF]

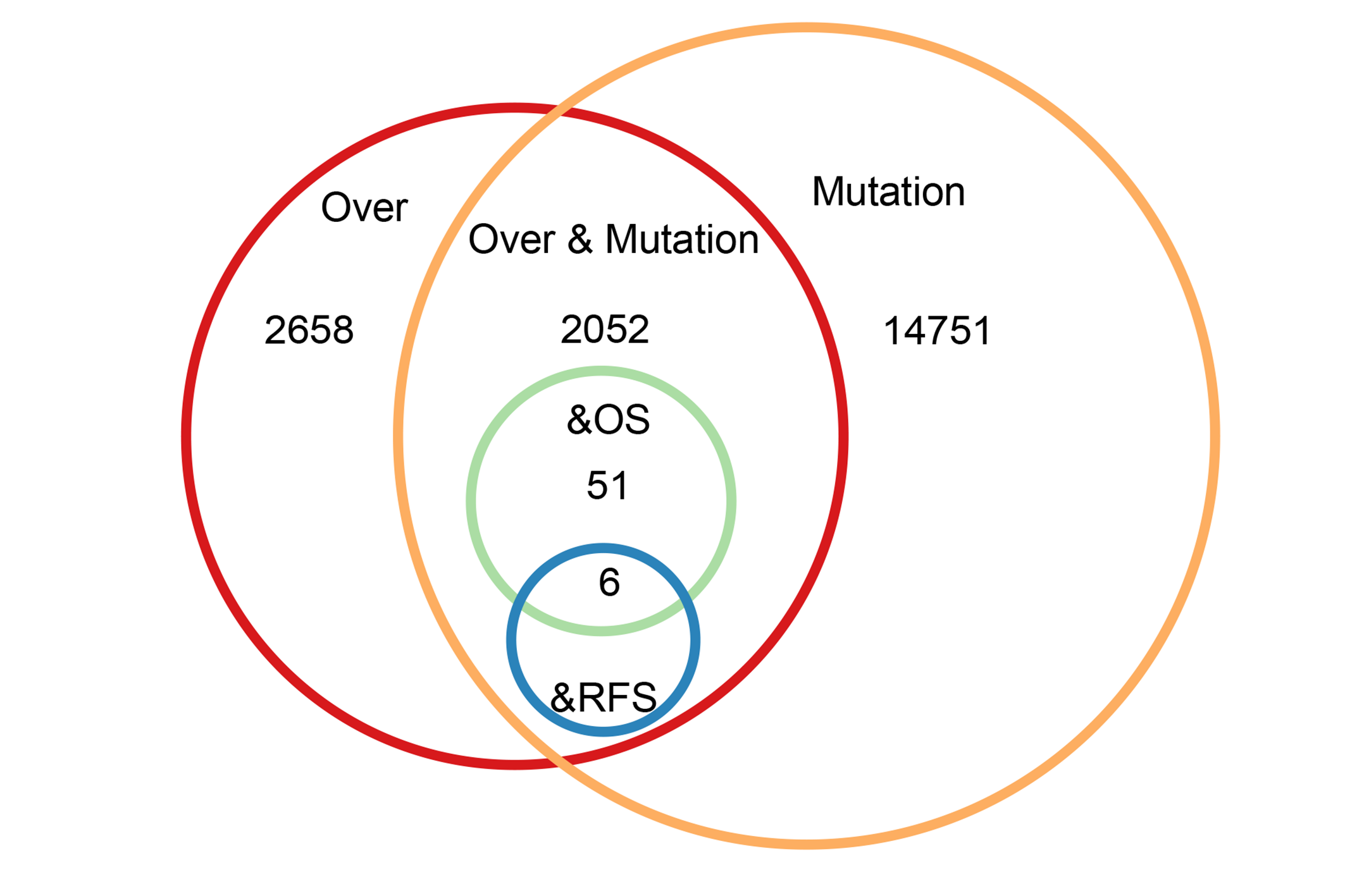

Supplement: Supplementary file 2 [file Image1.TIF]
