# Supplementary material for: Identification of Tumor Antigens and Design of mRNA Vaccine for Colorectal Cancer Based on the Immune Subtype
Source: Front Cell Dev Biol. 2022 Jan 20;9:783527. doi: 10.3389/fcell.2021.783527 (PMC8811447; doi:10.3389/fcell.2021.783527)
Supplement: Supplementary file 3 [file DataSheet1.docx]

Supplementary Material

# Supplementary Figures

**
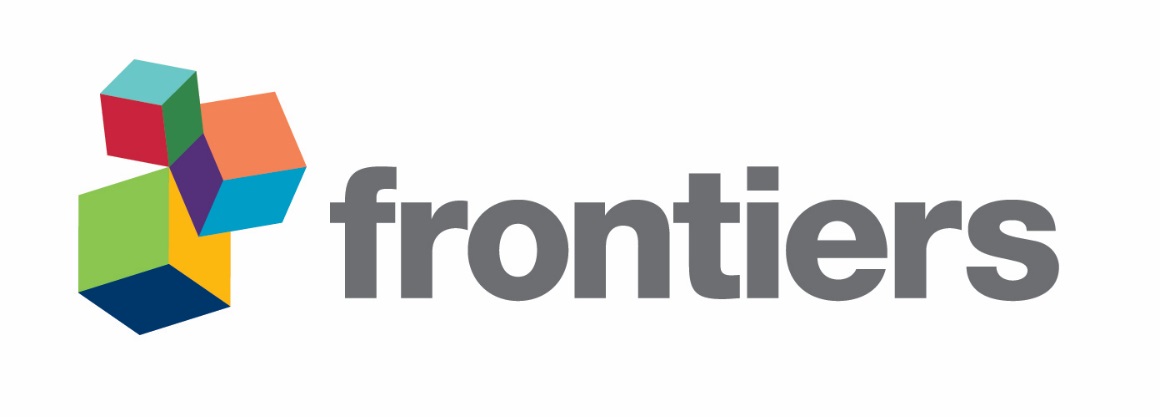
**

**Supplementary Figure 1.** Venn diagram for the selection of tumor antigens in CRC.

**Supplementary Figure 2.** Correlation of tumor antigens with CRC prognosis. (A-F) The association ofTHBS2 (A), FSTL3 (B), TNNT1(C), BGN (D), CTHRC1 (E), NOX4 (F) expression levels with the RFS of CRC patients.

THBS2 DNA sequence : ACGGCATCCAGTACAGAGGGGCTGGACTTGGACCCCTGCAGCAGCCCTGCACAGGAGAAGCGGCATATAAAGCCGCGCTGCCCGGGAGCCGCTCGGCCACGTCCACCGGAGCATCCTGCACTGCAGGGCCGGTCTCTCGCTCCAGCAGAGCCTGCGCCTTTCTGACTCGGTCCGGAACACTGAAACCAGTCATCACTGCATCTTTTTGGCAAACCAGGAGCTCAGCTGCAGGAGGCAGGATGGTCTGGAGGCTGGTCCTGCTGGCTCTGTGGGTGTGGCCCAGCACGCAAGCTGGTCACCAGGACAAAGACACGACCTTCGACCTTTTCAGTATCAGCAACATCAACCGCAAGACCATTGGCGCCAAGCAGTTCCGCGGGCCCGACCCCGGCGTGCCGGCTTACCGCTTCGTGCGCTTTGACTACATCCCACCGGTGAACGCAGATGACCTCAGCAAGATCACCAAGATCATGCGGCAGAAGGAGGGCTTCTTCCTCACGGCCCAGCTCAAGCAGGACGGCAAGTCCAGGGGCACGCTGTTGGCTCTGGAGGGCCCCGGTCTCTCCCAGAGGCAGTTCGAGATCGTCTCCAACGGCCCCGCGGACACGCTGGATCTCACCTACTGGATTGACGGCACCCGGCATGTGGTCTCCCTGGAGGACGTCGGCCTGGCTGACTCGCAGTGGAAGAACGTCACCGTGCAGGTGGCTGGCGAGACCTACAGCTTGCACGTGGGCTGCGACCTCATAGGACCAGTTGCTCTGGACGAGCCCTTCTACGAGCACCTGCAGGCGGAAAAGAGCCGGATGTACGTGGCCAAAGGCTCTGCCAGAGAGAGTCACTTCAGGGGTTTGCTTCAGAACGTCCACCTAGTGTTTGAAAACTCTGTGGAAGATATTCTAAGCAAGAAGGGTTGCCAGCAAGGCCAGGGAGCTGAGATCAACGCCATCAGTGAGAACACAGAGACGCTGCGCCTGGGTCCGCATGTCACCACCGAGTACGTGGGCCCCAGCTCGGAGAGGAGGCCCGAGGTGTGCGAACGCTCGTGCGAGGAGCTGGGAAACATGGTCCAGGAGCTCTCGGGGCTCCACGTCCTCGTGAACCAGCTCAGCGAGAACCTCAAGAGAGTGTCGAATGATAACCAGTTTCTCTGGGAGCTCATTGGTGGCCCTCCTAAGACAAGGAACATGTCAGCTTGCTGGCAGGATGGCCGGTTCTTTGCGGAAAATGAAACGTGGGTGGTGGACAGCTGCACCACGTGTACCTGCAAGAAATTTAAAACCATTTGCCACCAAATCACCTGCCCGCCTGCAACCTGCGCCAGTCCATCCTTTGTGGAAGGCGAATGCTGCCCTTCCTGCCTCCACTCGGTGGACGGTGAGGAGGGCTGGTCTCCGTGGGCAGAGTGGACCCAGTGCTCCGTGACGTGTGGCTCTGGGACCCAGCAGAGAGGCCGGTCCTGTGACGTCACCAGCAACACCTGCTTGGGGCCCTCGATCCAGACACGGGCTTGCAGTCTGAGCAAGTGTGACACCCGCATCCGGCAGGACGGCGGCTGGAGCCACTGGTCACCTTGGTCTTCATGCTCTGTGACCTGTGGAGTTGGCAATATCACACGCATCCGTCTCTGCAACTCCCCAGTGCCCCAGATGGGGGGCAAGAATTGCAAAGGGAGTGGCCGGGAGACCAAAGCCTGCCAGGGCGCCCCATGCCCAATCGATGGCCGCTGGAGCCCCTGGTCCCCGTGGTCGGCCTGCACTGTCACCTGTGCCGGTGGGATCCGGGAGCGCACCCGGGTCTGCAACAGCCCTGAGCCTCAGTACGGAGGGAAGGCCTGCGTGGGGGATGTGCAGGAGCGTCAGATGTGCAACAAGAGGAGCTGCCCCGTGGATGGCTGTTTATCCAACCCCTGCTTCCCGGGAGCCCAGTGCAGCAGCTTCCCCGATGGGTCCTGGTCATGCGGCTTCTGCCCTGTGGGCTTCTTGGGCAATGGCACCCACTGTGAGGACCTGGACGAGTGTGCCCTGGTCCCCGACATCTGCTTCTCCACCAGCAAGGTGCCTCGCTGTGTCAACACTCAGCCTGGCTTCCACTGCCTGCCCTGCCCGCCCCGATACAGAGGGAACCAGCCCGTCGGGGTCGGCCTGGAAGCAGCCAAGACGGAAAAGCAAGTGTGTGAGCCCGAAAACCCATGCAAGGACAAGACACACAACTGCCACAAGCACGCGGAGTGCATCTACCTGGGTCACTTCAGCGACCCCATGTACAAGTGCGAGTGCCAGACAGGCTACGCGGGCGACGGGCTCATCTGCGGGGAGGACTCGGACCTGGACGGCTGGCCCAACCTCAATCTGGTCTGCGCCACCAACGCCACCTACCACTGCATCAAGGATAACTGCCCCCATCTGCCAAATTCTGGGCAGGAAGACTTTGACAAGGACGGGATTGGCGATGCCTGTGATGATGACGATGACAATGACGGTGTGACCGATGAGAAGGACAACTGCCAGCTCCTCTTCAATCCCCGCCAGGCTGACTATGACAAGGATGAGGTTGGGGACCGCTGTGACAACTGCCCTTACGTGCACAACCCTGCCCAGATCGACACAGACAACAATGGAGAGGGTGACGCCTGCTCCGTGGACATTGATGGGGACGATGTCTTCAATGAACGAGACAATTGTCCCTACGTCTACAACACTGACCAGAGGGACACGGATGGTGACGGTGTGGGGGATCACTGTGACAACTGCCCCCTGGTGCACAACCCTGACCAGACCGACGTGGACAATGACCTTGTTGGGGACCAGTGTGACAACAACGAGGACATAGATGACGACGGCCACCAGAACAACCAGGACAACTGCCCCTACATCTCCAACGCCAACCAGGCTGACCATGACAGAGACGGCCAGGGCGACGCCTGTGACCCTGATGATGACAACGATGGCGTCCCCGATGACAGGGACAACTGCCGGCTTGTGTTCAACCCAGACCAGGAGGACTTGGACGGTGATGGACGGGGTGATATTTGTAAAGATGATTTTGACAATGACAACATCCCAGATATTGATGATGTGTGTCCTGAAAACAATGCCATCAGTGAGACAGACTTCAGGAACTTCCAGATGGTCCCCTTGGATCCCAAAGGGACCACCCAAATTGATCCCAACTGGGTCATTCGCCATCAAGGCAAGGAGCTGGTTCAGACAGCCAACTCGGACCCCGGCATCGCTGTAGGTTTTGACGAGTTTGGGTCTGTGGACTTCAGTGGCACATTCTACGTAAACACTGACCGGGACGACGACTATGCTGGCTTCGTCTTTGGTTACCAGTCAAGCAGCCGCTTCTATGTGGTGATGTGGAAGCAGGTGACGCAGACCTACTGGGAGGACCAGCCCACGCGGGCCTATGGCTACTCCGGCGTGTCCCTCAAGGTGGTGAACTCCACCACGGGGACGGGCGAGCACCTGAGGAACGCGCTGTGGCACACGGGGAACACGCCGGGGCAGGTGCGAACCTTATGGCACGACCCCAGGAACATTGGCTGGAAGGACTACACGGCCTATAGGTGGCACCTGACTCACAGGCCCAAGACCGGCTACATCAGAGTCTTAGTGCATGAAGGAAAACAGGTCATGGCAGACTCAGGACCTATCTATGACCAAACCTACGCTGGCGGGCGGCTGGGTCTATTTGTCTTCTCTCAAGAAATGGTCTATTTCTCAGACCTCAAGTACGAATGCAGAGATATTTAAACAAGATTTGCTGCATTTCCGGCAATGCCCTGTGCATGCCATGGTCCCTAGACACCTCAGTTCATTGTGGTCCTTGCGGCTTCTCTCTCTAGCAGCACCTCCTGTCCCTTGACCTTAACTCTGATGGTTCTTCACCTCCTGCCAGCAACCCCAAACCCAAGTGCCTTCAGAGGATAAATATCAATGGAACTCAGAGATGAACATCTAACCCACTAGAGGAAACCAGTTTGGTGATATATGAGACTTTATGTGGAGTGAAAATTGGGCATGCCATTACATTGCTTTTTCTTGTTTGTTTAAAAAGAATGACGTTTACATATAAAATGTAATTACTTATTGTATTTATGTGTATATGGAGTTGAAGGGAATACTGTGCATAAGCCATTATGATAAATTAAGCATGAAAAATATTGCTGAACTACTTTTGGTGCTTAAAGTTGTCACTATTCTTGAATTAGAGTTGCTCTACAATGACACACAAATCCCGCTAAATAAATTATAAACAAGGGTCAATTCAAATTTGAAGTAATGTTTTAGTAAGGAGAGATTAGAAGACAACAGGCATAGCAAATGACATAAGCTACCGATTAACTAATCGGAACATGTAAAACAGTTACAAAAATAAACGAACTCTCCTCTTGTCCTACAATGAAAGCCCTCATGTGCAGTAGAGATGCAGTTTCATCAAAGAACAAACATCCTTGCAAATGGGTGTGACGCGGTTCCAGATGTGGATTTGGCAAAACCTCATTTAAGTAAAAGGTTAGCAGAGCAAAGTGCGGTGCTTTAGCTGCTGCTTGTGCCGTTGTGGCGTCGGGGAGGCTCCTGCCTGAGCTTCCTTCCCCAGCTTTGCTGCCTGAGAGGAACCAGAGCAGACGCACAGGCCGGAAAAGGCGCATCTAACGCGTATCTAGGCTTTGGTAACTGCGGACAAGTTGCTTTTACCTGATTTGATGATACATTTCATTAAGGTTCCAGTTATAAATATTTTGTTAATATTTATTAAGTGACTATAGAATGCAACTCCATTTACCAGTAACTTATTTTAAATATGCCTAGTAACACATATGTAGTATAATTTCTAGAAACAAACATCTAATAAGTATATAATCCTGTGAAAATATGAGGCTTGATAATATTAGGTTGTCACGATGAAGCATGCTAGAAGCTGTAACAGAATACATAGAGAATAATGAGGAGTTTATGATGGAACCTTAATATATAATGTTGCCAGCGATTTTAGTTCAATATTTGTTACTGTTATCTATCTGCTGTATATGGAATTCTTTTAATTCAAACGCTGAAAACGAATCAGCATTTAGTCTTGCCAGGCACACCCAATAATCAGTCATGTGTAATATGCACAAGTTTGTTTTTGTTTTTGTTTTTTTTGTTGGTTGGTTTTTTTGCTTTAAGTTGCATGATCTTTCTGCAGGAAATAGTCACTCATCCCACTCCACATAAGGGGTTTAGTAAGAGAAGTCTGTCTGTCTGATGATGGATAGGGGGCAAATCTTTTTCCCCTTTCTGTTAATAGTCATCACATTTCTATGCCAAACAGGAACGATCCATAACTTTAGTCTTAATGTACACATTGCATTTTGATAAAATTAATTTTGTTGTTTCCTTTGAGGTTGATCGTTGTGTTGTTTTGCTGCACTTTTTACTTTTTTGCGTGTGGAGCTGTATTCCCGAGACAACGAAGCGTTGGGATACTTCATTAAATGTAGCGACTGTCAACAGCGTGCAGGTTTTCTGTTTCTGTGTTGTGGGGTCAACCGTACAATGGTGTGGGAATGACGATGATGTGAATATTTAGAATGTACCATATTTTTTGTAAATTATTTATGTTTTTCTAAACAAATTTATCGTATAGGTTGATGAAACGTCATGTGTTTTGCCAAAGACTGTAAATATTTATTTATGTGTTCACATGGTCAAAATTTCACCACTGAAACCCTGCACTTAGCTAGAACCTCATTTTTAAAGATTAACAACAGGAAATAAATTGTAAAAAAGGTTTTCT

FSTL3 DNA sequence**:** AAGTCGGTGCCGCTGCCGTCTCTGCGTTCGCCATGCGTCCCGGGGCGCCAGGGCCACTCTGGCCTCTGCCCTGGGGGGCCCTGGCTTGGGCCGTGGGCTTCGTGAGCTCCATGGGCTCGGGGAACCCCGCGCCCGGTGGTGTTTGCTGGCTCCAGCAGGGCCAGGAGGCCACCTGCAGCCTGGTGCTCCAGACTGATGTCACCCGGGCCGAGTGCTGTGCCTCCGGCAACATTGACACCGCCTGGTCCAACCTCACCCACCCGGGGAACAAGATCAACCTCCTCGGCTTCTTGGGCCTTGTCCACTGCCTTCCCTGCAAAGATTCGTGCGACGGCGTGGAGTGCGGCCCGGGCAAGGCGTGCCGCATGCTGGGGGGCCGCCCGCGCTGCGAGTGCGCGCCCGACTGCTCGGGGCTCCCGGCGCGGCTGCAGGTCTGCGGCTCAGACGGCGCCACCTACCGCGACGAGTGCGAGCTGCGCGCCGCGCGCTGCCGCGGCCACCCGGACCTGAGCGTCATGTACCGGGGCCGCTGCCGCAAGTCCTGTGAGCACGTGGTGTGCCCGCGGCCACAGTCGTGCGTCGTGGACCAGACGGGCAGCGCCCACTGCGTGGTGTGTCGAGCGGCGCCCTGCCCTGTGCCCTCCAGCCCCGGCCAGGAGCTTTGCGGCAACAACAACGTCACCTACATCTCCTCGTGCCACATGCGCCAGGCCACCTGCTTCCTGGGCCGCTCCATCGGCGTGCGCCACGCGGGCAGCTGCGCAGGCACCCCTGAGGAGCCGCCAGGTGGTGAGTCTGCAGAAGAGGAAGAGAACTTCGTGTGAGCCTGCAGGACAGGCCTGGGCCTGGTGCCCGAGGCCCCCCATCATCCCCTGTTATTTATTGCCACAGCAGAGTCTAATTTATATGCCACGGACACTCCTTAGAGCCCGGATTCGGACCACTTGGGGATCCCAGAACCTCCCTGACGATATCCTGGAAGGACTGAGGAAGGGAGGCCTGGGGGCCGGCTGGTGGGTGGGATAGACCTGCGTTCCGGACACTGAGCGCCTGATTTAGGGCCCTTCTCTAGGATGCCCCAGCCCCTACCCTAAGACCTATTGCCGGGGAGGATTCCACACTTCCGCTCCTTTGGGGATAAACCTATTAATTATTGCTACTATCAAGAGGGCTGGGCATTCTCTGCTGGTAATTCCTGAAGAGGCATGACTGCTTTTCTCAGCCCCAAGCCTCTAGTCTGGGTGTGTACGGAGGGTCTAGCCTGGGTGTGTACGGAGGGTCTAGCCTGGGTGAGTACGGAGGGTCTAGCCTGGGTGAGTACGGAGGGTCTAGCCTGGGTGAGTACGGAGGGTCTAGCCTGGGTGTGTATGGAGGATCTAGCCTGGGTGAGTATGGAGGGTCTAGCCTGGGTGAGTATGGAGGGTCTAGCCTGGGTGTGTATGGAGGGTCTAGCCTGGGTGAGTATGGAGGGTCTAGCCTGGGTGTGTATGGAGGGTCTAGCCTGGGTGAGTATGGAGGGTCTAGCCTGGGTGTGTACGGAGGGTCTAGTCTGAGTGCGTGTGGGGACCTCAGAACACTGTGACCTTAGCCCAGCAAGCCAGGCCCTTCATGAAGGCCAAGAAGGCTGCCACCATTCCCTGCCAGCCCAAGAACTCCAGCTTCCCCACTGCCTCTGTGTGCCCCTTTGCGTCCTGTGAAGGCCATTGAGAAATGCCCAGTGTGCCCCCTGGGAAAGGGCACGGCCTGTGCTCCTGACACGGGCTGTGCTTGGCCACAGAACCACCCAGCGTCTCCCCTGCTGCTGTCCACGTCAGTTCATGAGGCAACGTCGCGTGGTCTCAGACGTGGAGCAGCCAGCGGCAGCTCAGAGCAGGGCACTGTGTCCGGCGGAGCCAAGTCCACTCTGGGGGAGCTCTGGCGGGGACCACGGGCCACTGCTCACCCACTGGCCCCGAGGGGGGTGTAGACGCCAAGACTCACGCATGTGTGACATCCGGAGTCCTGGAGCCGGGTGTCCCAGTGGCACCACTAGGTGCCTGCTGCCTCCACAGTGGGGTTCACACCCAGGGCTCCTTGGTCCCCCACAACCTGCCCCGGCCAGGCCTGCAGACCCAGACTCCAGCCAGACCTGCCTCACCCACCAATGCAGCCGGGGCTGGCGACACCAGCCAGGTGCTGGTCTTGGGCCAGTTCTCCCACGACGGCTCACCCTCCCCTCCATCTGCGTTGATGCTCAGAATCGCCTACCTGTGCCTGCGTGTAAACCACAGCCTCAGACCAGCTATGGGGAGAGGACAACACGGAGGATATCCAGCTTCCCCGGTCTGGGGTGAGGAATGTGGGGAGCTTGGGCATCCTCCTCCAGCCTCCTCCAGCCCCCAGGCAGTGCCTTACCTGTGGTGCCCAGAAAAGTGCCCCTAGGTTGGTGGGTCTACAGGAGCCTCAGCCAGGCAGCCCACCCCACCCTGGGGCCCTGCCTCACCAAGGAAATAAAGACTCAA

TNNT1 DNA sequence: GCTGTACAACCGCATCAGCCACGCCCAGAAGTTGTGAGTGTGACCGTCTCAAGCCCTTGCTCTTGTCCCTACCCTCCCGCTGACTCTCAGGCCTCTTTCCCTCCCTCTGTGGGCTCCATCCCCCACTGCCTGAGTTTCTGCCCCTTCTCTTTGTCTTCTTCGTCTGTCTCTCTCTCTGCCTCTGGAAATCTGGGCATGTTCTTTCCCTTTCCCTTTCCCTTTCCCTTTTTTTTTTTTTTATTGAGACTGCGTCTCGCTCTGTCACCCAGGCTGGAGTGCAGTGGAGTGATCTCAGCTCACTGCAACTTCCATGGGCCCTCTTCAAGCGATTCTCCTGCCTCAGCCCCCTGAGTAGCTGGGATTACAGGTGCGCCCGCACCAAGCGTNNNNNNNNNNNNNNNNNNNNNNNNNNNNNNNNNNNNNNNNNNNNNNNNNNNNNNNNNNNNNNNNNNNNNNNNNNNNNNNNNNNNNNNNNNNNNNNNNNNNTCGAACTCCTGACCTCGTGATCCACCCGCCTTGGCCTGGTAAAGTGCTGGGATTACAGGCGTGACGACCGCGCCCAGCCGAGAGGGTTCCCTGAATCTCTCTGTTTCTCCACTCCCTCTTACGCTTCCCTCCTCTCCTCTCCCTTCTCCTCTCTCCCATTTCTGTCTCTTTCCAGCCCTTCTGCGTGACCCATGCCCAGATATCCATACGCTATAACATTCATCAGCTCCCATGAACACCCTGACTGNCCCAAATATCAGTNCCACATGTCAGCGAGGCTTTGACCACCAGCCGCTGCCCCCCAGTCATAGACCCAAAAGAGGTACAGTTGTTTTGCTGAGTGCTTCCNTTTNNGGGAGGGCTAATATTTTGGGAATGGGCTTGGTGAAGGTTGAGCCTGGGGCCCATTTCAGAGACTGGAGGGGCCCCTTTTTCCTGACTACCACCTCATTCTTCTACAGCCGGAAGGGGGCAGGGAAGGGCCGCGTTGGAGGCCGCTGGAAGTGAGGATGCCGCCCCGGACAGTGGCACCTGGGAAGCCTGGGAGTGTTTGTCCCATCG

BGN DNA sequence:

GATCCTGGAGAAACCACCTCCTTGCTTAGGCCCAAGCAGGTTCCTGGCAGGCTCAGGACCAAATTCCAGGGGCCACTCATGGGCCTAGCAGCCCAAGGCCGCCTCCCCCTCGTCTTTCTTCCATCTCTCTTTCCTCGTCCTGGCGAGATGCCAGCCAGCACCTCAGTGTCCCCATCTGGGCAGTGGAAAGTTTGACTCTCTGGGTCCTTGTTTGAGTGAGTGCGAGTGTGTCCGTTCCTTTGCTGTCTGCCCCAGGCGGGGGAGGGGGGGGGAGGTGGTGGGGGCGAGGGGGCGGGGGCTCAGCTAGTCCAGCCGTCTACAAGAAAATTGCTCCCTTTGAAGCTGCCAGGGGGGCCGGGAAGCCTGCCCCCTCCTGCTCGCCCGCCCTCTCCGCCCCACCAGCCCCCTCCCTCCTTTCCTCCCTCCCCGCCCTCTCCCCGCTGTCCCCTCCCCGTCGGCCCGCCTGCCCAGCCTTTAGCCTCCCGCCCGCCGCCTCTGTCTCCCTCTCTCCACAAACTGCCCAGGAGTGAGTAGCTGCTTTCGGTCCGCCGGACACACCGGACAGATAGACGTGCGGACGGCCCACCACCCCAGCCCGCCAACTAGTCAGCCTGCGCCTGGCGCCTCCCCTCTCCAGGTAGGGCTGGCTTCAAGCTGCCTCCTCAGCAACCCAGAGATGCCCCTGGCTCTGCTGCCTCCGCTGTCCCAAGCCCTGGTCCTGCTGTCCCCAGTGCCGCGAGGGTGTCCACAGATTTCCCCGGTGCTCTCTGTAGGCTGCTGATCCACGCCCCTTCATCGCCACCCTGCGGCCCCCTTGGTCCCTGTCAGGCTTCTGCTCGTCTCGCCGCCCTCCAGGCACCTTTCCCTCACCCCTTCCTCTCCCTTCTGACCTTGCTCTGCTTCATCCACCTCTTGTCTCTCTGCCTCCCACTCGGGGTCCGTCTTCTTGGCTACCACCCTAGAGCGTGGCTGGGTGACTGGTACCCCAGCTTTGCCAATGGCCCTGTTTCATCATTGCAAGTCCCAGGCGCATGCTCCACTCCCTCAGCCTCGCTCTGCCCAGGCGCCTCCTTGCTCCAGGCTTGGCGCCTGGCCCGGGTTGGGTCGGATCGGGGAGGACCGCCCAGCGCCCACCGAGCTCNNNNNNNNNNNNNNNNNNNNNNNNNNNNNNNNNNNNNNNNNNNNNNNNNNNNNNNNNNNNNNNNNNNNNNNNNNNNNNNNNNNNNNNNNNNNNNNNNNNNACAGGTGGGTGCTGGTGCTGATGATCCCCTCGCCTCTTCCCCCAGGTCCATCCGCCATGTGGCCCCTGTGGCGCCTCGTGTCTCTGCTGGCCCTGAGCCAGGCCCTGCCCTTTGAGCAGAGAGGCTTCTGGGACTTCACCCTGGACGATGGGCCATTCATGATGAACGATGAGGAAGCTTCGGGCGCTGACACCTCGGGCGTCCTGGACCCGGACTCTGTCACACCCACCTACAGCGCCATGTGTCCTTTCGGCTGCCACTGCCACCTGCGGGTGGTTCAGTGCTCCGACCTGGGTTTGTCCCTGAGTGATGGGGAGCGGGGCATGCAGGGAGGCTCAGGTGCAGCCTGAGAGCCCCTTCTGAAGGGGGCACATGCTGGTCCTGTGGACGGTGGCGAGCATGATGTAAGTGTAGGAGGGGTCCAGCCGTCTGGCTGTGAGCTGTGCAGTTTGTGCCCACTTGTGGTGGCATCCCCGTGTGCCCGTCAGTGTCCCTGTGTGTGTGTCCCCGGTCCTCCCTACCAGTGGGGCTAGTCGGCTGGATGGCTCCAAGTTCATGCTGGTGATGGTGGTGGGGCCCCTAGGTCTCGAGTTCATGCTGGTGGTGGGGGTGGGGCCCCTAGGTCTCAAGTTCATGCTGGTGATGGGGGTGGGGCCCCTAGGTCTGAAGTCTGTGCCCAAAGAGATCTCCCCTGACACCACGCTGCTGGACCTGCAGAACAACGACATCTCCGAGCTCCGCAAGGATGACTTCAAGGGTCTCCAGCACCTCTACGTAAGGAGCTGGGAGGAACCAGCAGGCCTACAGCAGAGGGCAGGGGTCCGGGTGGGTGCATGTGCGTGGACGTGTGGGGTATGAGAGGGGTTCGGGGACTCGTGGGACTTCAGGGTGAAGCCTGGAGCCAGCCGTGATGGGAGCTCCCGGGTTTGCGGCTCACTCATGTGGGTTTGAGCAACCACAGCTGCAGGACCGGATCGCTCAGTTCGGCTCCCTTCGTGGCTGAAAACGTTTCATCACGTCCACTCCTCCCAGCAACAGAGGAGAACGGATTTCATTGTAGCCAGTGTGCGTGTGAGGAAACTGAGGCTGGGAGCGGCAAGGCAGTGGTGGCACTGCTGGGGCTCAGGACCGGGCCTGGGTGCTGCCTCCTGCCCTGCACTCTGCTCACAAGCATGGACTGACCTCCTCGAGCGCCAGTGGGCTGGGGAGGCACAGGAAGGCAGGAGAGAGGGGCGGGTGGGGTGGGGAGTCTGTGCCTTCACCTCCTCCGCCCACCCTGCTTCAGGCCCTCGTCCTGGTGAACAACAAGATCTCCAAGATCCATGAGAAGGCCTTCAGCCCACTGCGGAACGTGCAGAAGCTCTACATCTCCAAGAACCACCTGGTGGAGATCCCGCCCAACCTACCCAGCTCCCTGGTGGAGCTCCGCATCCACGACAACCGCATCCGCAAGGTGCCCAAGGGAGTGTTCAGTGGGCTCCGGAACATGAACTGCATCGGTGAGCTGAGGGCCTCCCAGAACATTCCAGAGCCTTGTCTCGAGGCATGGGGAAGGGAGACCAAGGAATACCTTTAGAGGCTCAGTTCAAGAAAGAGTATGGTGAGAACGGTCAAAAGAAAATCCATGGATTTCTTGGCAAATCCTCCATGCAGGCGATCACCACGGCTAAAGAGAAGACTGGCCAGAGGGGCCGGGTGGCTTCCGGAGCCCCATCTTCATCTCTGGCACTCCTCCCTTTCCTCTTGCTGCCCCTGGAGCTAGCAGTCCTGGGGCTAGCAGTCCTGAACAGCTAGGAGTTTGCAATTAGCCCGGTAAATTAGCAGAACTGCTTTCAGGAGACGGGAGCAGCCGGCAGGTAGCAGGGCCCACCACACTGGCCCGGAAGTGACAGGACCCAGGGCTGTGCAGGGACCACCAGGCTCCCGGGCTAATGAGGTCTCTCCCCTAGAGATGGGCGGGAACCCACTGGAGAACAGTGGCTTTGAACCTGGAGCCTTCGATGGCCTGAAGCTCAACTACCTGCGCATCTCAGAGGCCAAGCTGACTGGCATCCCCAAAGGTAGGAAGCCCACTCTTCCTGCACGCCTGCCTGCCTCACCCCCAACAGCACAGATGGCCAGGGTGGGGGCTCTGGATGGGCCCGATCTACTCAGGGAAAGGCTCAACAGTCCCCTCCCGCCACCTGGGGCAGAGCTAGGGCCCCTGCCCTCAGCACCTGCATTCTCCCCTGTGCCCTCTTCTCCTGGCAGACCTCCCTGAGACCCTGAATGAACTCCACCTAGACCACAACAAAATCCAGGCCATCGAACTGGAGGACCTGCTTCGCTACTCCAAGCTGTACAGGTGAGGCCAGCAGGGCACCGCCAAGGGTGATGCCAGAGTCCCTCAGTGCTGTGTGGCCCCTCGCGCCCAGCCCCCCATCCTTACCTCCAGCCTTTGAGTCCGTGTCATTCTCCCGCTCACAGGCTGGGCCTAGGCCACAACCAGATCAGGATGATCGAGAACGGGAGCCTGAGCTTCCTGCCCACCCTCCGGGAGCTCCACTTGGACAACAACAAGTTGGCCAGGGTGCCCTCAGGGCTCCCAGACCTCAAGCTCCTCCAGGTGAGAGCTGGGCATGCACAGCCAGGNNNNNNNNNNNNNNNNNNNNNNNNNNNNNNNNNNNNNNNNNNNNNNNNNNNNNNNNNNNNNNNNNNNNNNNNNNNNNNNNNNNNNNNNNNNNNNNNNNNNACCTCACACCACCAAACACACCTCTACCCCAGCCCCGCCCCCACATGTCCTCAACCTGACCCACCTGAGACCCTCATCCTTGTCCCTGGTCACATCCAGTGCCTTAATCCTGGCTGACACCCACACAAATAACACGCCCATGCCTTGGTTTGCTCCTCCCAACAACGGGGAGCCTCTGGTGTGGCCCTTGAAGTAGGTTGCAGAGGCAACAGCAAAATGCCTCCTGGAGGCAGCGGGCTTGGCGTGGAGGGAGGGAGGCCTGTGACCCGGCCTCTCTGCCTTCAGGTGGTCTATCTGCACTCCAACAACATCACCAAAGTGGGTGTCAACGACTTCTGTCCCATGGGCTTCGGGGTGAAGCGGGCCTACTACAACGGCATCAGCCTCTTCAACAACCCCGTGCCCTACTGGGAGGTGCAGCCGGCCACTTTCCGCTGCGTCACTGACCGCCTGGCCATCCAGTTTGGCAACTACAAAAAGTAGAGGCAGCTGCAGCCACCGCGGGGCCTCAGTGGGGGTCTCTGGGGAACACAGCCAGACATCCTGATGGGGAGGCAGAGCCAGGAAGCTAAGCCAGGGCCCAGCTGCGTCCAACCCAGCCCCCCACCTCAGGTCCCTGACCCCAGCTCGATGCCCCATCACCGCCTCTCCCTGGCTCCCAAGGGTGCAGGTGGGCGCAAGGCCCGGCCCCCATCACATGTTCCCTTGGCCTCAGAGCTGCCCCTGCTCTCCCACCACAGCCACCCAGAGGCACCCCATGAAGCTTTTTTCTCGTTCACTCCCAAACCCAAGTGTCCAAAGCTCCAGTCCTAGGAGAACAGTCCCTGGGTCAGCAGCCAGGAGGCGGTCCATAAGAATGGGGACAGTGGGCTCTGCCAGGGCTGCCGCACCTGTCCAGAACAACATGTTCTGTTCCTCCTCCTCATGCATTTCCAGCCTTGNNNNNNNNNNNNNNNNNNNNNNNNNNNNNNNNNNNNNNNNNNNNNNNNNNNNNNNNNNNNNNNNNNNNNNNNNNNNNNNNNNNNNNNNNNNNNNNNNNNNGGACAGCGGTCTCCCCAGCCTGCCCTGCTCAGCCCTGCCCCCAAACCTGTACTGTCCCGGAGGAGGTTGGGAGGTGGAGGCCCAGCATCCCGCGCAGATGACACCATCAACCGCCAGAGTCCCAGACACCGGTTTTCCTAGAAGCCCCTCACCCCCACTGGCCCACTGGTGGCTAGGTCTCCCCTTACTCTTCTGGTCCAGCGCAACCAGGGGCTGCTTCTGAGGTCGGTGGCTGTCTTTCCATTAAAGAAACACCGTGC

CTHRC1 DNA sequence: GGCACGAGGGCGGCCTCGGAGCGCGGCGGAGCCAGACGCTGACCACGTTCCTCTCCTCGGTCTCCTCCGCCTCCAGCTCCGCGCTGCCCGGCAGCCGGGAGCCATGCGACCCCAGGGCCCCGCCGCCTCCCCGCAGCGGCTCCGCGGCCTCCTGCTGCTCCTGCTGCTGCAGCTGCCCGCGCCGTCGAGCGCCTCTGAGATCCCCAAGGGGAAGCAAAAGGCGCAGCTCCGGCAGAGGGAGGTGGTGGACCTGTATAATGGAATGTGCTTACAAGGGCCAGCAGGAGTGCCTGGTCGAGACGGGAGCCCTGGGGCCAATGGCATTCCGGGTACACCTGGGATCCCAGGTCGGGATGGATTCAAAGGAGAAAAGGGGGAATGTCTGAGGGAAAGCTTTGAGGAGTCCTGGACACCCAACTACAAGCAGTGTTCATGGAGTTCATTGAATTATGGCATAGATCTTGGGAAAATTGCGGAGTGTACATTTACAAAGATGCGTTCAAATAGTGCTCTAAGAGTTTTGTTCAGTGGCTCACTTCGGCTAAAATGCAGAAATGCATGCTGTCAGCGTTGGTATTTCACATTCAATGGAGCTGAATGTTCAGGACCTCTTCCCATTGAAGCTATAATTTATTTGGACCAAGGAAGCCCTGAAATGAATTCAACAATTAATATTCATCGCACTTCTTCTGTGGAAGGACTTTGTGAAGGAATTGGTGCTGGATTAGTGGATGTTGCTATCTGGGTTGGCACTTGTTCAGATTACCCAAAAGGAGATGCTTCTACTGGATGGAATTCAGTTTCTCGCATCATTATTGAAGAACTACCAAAATAAATGCTTTAATTTTCATTTGCTACCTCTTTTTTTATTATGCCTTGGAATGGTTCACTTAAATGACATTTTAAATAAGTTTATGTATACATCTGAATGAAAAGCAAAGCTAAATATGTTTACAGACCAAAGTGTGATTTCACACTGTTTTTAAATCTAGCATTATTCATTTTGCTTCAATCAAAAGTGGTTTCAATATTTTTTTAGTTGGTTAGAATACTTTCTTCATAGTCACATTCTCTCAACCTATAATTTGGAATATTGTTGTGGTCTTTTGTTTTTTCTCTTAGTATAGCATTTTTAAAAAAATATAAAAGCTACCAATCTTTGTACAATTTGTAAATGTTAAGAATTTTTTTTATATCTGTTAAATAAAAATTATTTCCAAC

NOX4 DNA sequence:

ATGGCTGTGTCCTGGAGGAGCTGGCTCGCCAACGAAGGGGTTAAACACCTCTGCCTGTTCATCTGGCTCTCCATGAATGTCCTGCTTTTCTGGAAAACCTTCTTGCTGTATAACCAAGGGCCAGAGTATCACTACCTCCACCAGATGTTGGGGCTAGGATTGTGTCTAAGCAGAGCCTCAGCATCTGTTCTTAACCTCAACTGCAGCCTTATCCTTTTACCCATGTGCCGAACACTCTTGGCTTACCTCCGAGGATCACAGAAGGTTCCAAGCAGGAGAACCAGGAGATTGTTGGATAAAAGCAGAACATTCCATATTACCTGTGGTGTTACTATCTGTATTTTCTCAGGCGTGCATGTGGCTGCCCATCTGGTGAATGCCCTCAACTTCTCAGTGAATTACAGTGAAGACTTTGTTGAACTGAATGCAGCAAGATACCGAGATGAGGATCCTAGAAAACTTCTCTTCACAACTGTTCCTGGCCTGACAGGGGTCTGCATGGTGGTGGTGCTATTCCTCATGATCACAGCCTCTACATATGCAATAAGAGTTTCTAACTATGATATCTTCTGGTATACTCATAACCTCTTCTTTGTCTTCTACATGCTGCTGACGTTGCATGTTTCAGGAGGGCTGCTGAAGTATCAAACTAATTTAGATACCCACCCTCCCGGCTGCATCAGTCTTAACCGAACCAGCTCTCAGAATATTTCCTTACCAGAGTATTTCTCAGAACATTTTCATGAACCTTTCCCTGAAGGATTTTCAAAACCGGCAGAGTTTACCCAGCACAAATTTGTGAAGATTTGTATGGAAGAGCCCAGATTCCAAGCTAATTTTCCACAGACTTGGCTTTGGATTTCTGGACCTTTGTGCCTGTACTGTGCCGAAAGACTTTACAGGTATATCCGGAGCAATAAGCCAGTCACCATCATTTCGGTCATAAGTCATCCCTCAGATGTCATGGAAATCCGAATGGTCAAAGAAAATTTTAAAGCAAGACCTGGTCAGTATATTACTCTACATTGTCCCAGTGTATCTGCATTAGAAAATCATCCATTTACCCTCACAATGTGTCCAACTGAAACCAAAGCAACATTTGGGGTTCATCTTAAAATAGTAGGAGACTGGACAGAACGATTTCGAGATTTACTACTGCCTCCATCTAGTCAAGACTCCGAAATTCTGCCCTTCATTCAATCTAGAAATTATCCCAAGGATGACTGGAAACCATACAAGCTTAGAAGACTATACTTTATTTGGGTATGCAGAGATATCCAGTCCTTCCGTTGGTTTGCAGATTTACTCTGTATGTTGCATAACAAGTTTTGGCAAGAGAACAGACCTGACTATGTCAACATCCAGCTGTACCTCAGTCAAACAGATGGGATACAGAAGATAATTGGAGAAAAATATCATGCACTGAATTCAAGACTGTTTATAGGACGTCCTCGGTGGAAACTTTTGTTTGATGAAATAGCAAAATATAACAGAGGAAAAACAGTTGGTGTTTTCTGTTGTGGACCCAATTCACTATCCAAGACTCTTCATAAACTGAGTAACCAGAACAACTCATATGGGACAAGATTTGAATACAATAAAGAGTCTTTCAGCTGA
